# Characterization and pathogenic mechanisms of a *Klebsiella aerogenes* strain isolated from a deceased ground thrush

**DOI:** 10.3389/fmicb.2026.1811142

**Published:** 2026-05-12

**Authors:** Mingao Sun, Yuxia Yang, Chelegeri Zhao, Bayi Erta, Wei Guo, Haifeng Wang, Rigetu Zhao, Luomeng Chao

**Affiliations:** 1 College of Animal Science and Technology, Inner Mongolia MINZU University, Tongliao, China; 2 College of Computer Science and Technology, Inner Mongolia MINZU University, Tongliao, China; 3 Tongliao Animal Agriculture Development Service Center, Tongliao, China; 4 Tongliao Animal Disease Control Center, Tongliao, China; 5 Tongliao Institute of Agricultural and Animal Husbandry Science, Tongliao, China; 6 Tongliao Animal Quarantine Technology Service Center, Tongliao, China; 7 Chifeng Academy of Agriculture and Animal Husbandry Sciences, Chifeng, China; 8 Inner Mongolia Rambo Testing Technology Limited Company, Tongliao, China; 9 Inner Mongolia Engineering Technology Research Center for Prevention and Control of Beef Cattle Diseases, Tongliao, Inner Mongolia, China; 10 Beef Cattle Industry School of Inner Mongolia Autonomous Region, lnner Mongolia MINZU University, Tongliao, China

**Keywords:** apoptosis, inflammatory response, *Klebsiella aerogenes*, pulmonary infection, signaling pathways, virulence genes

## Abstract

*Klebsiella aerogenes* is a significant opportunistic pathogen, yet the virulence and resistance mechanisms of wildlife-originating strains remain poorly understood. This study aimed to investigate the biological characteristics of *K. aerogenes* strain S_KLB, isolated from the lung of a deceased ground thrush, and to elucidate its pathogenic mechanisms in murine pneumonia. The isolated strain was assessed for cultural characteristics, antibiotic susceptibility, and the presence of virulence genes via PCR. An acute pneumonia model was established in mice through intranasal infection. Clinical symptoms and histopathological alterations in lung tissues were evaluated using Hematoxylin and Eosin (H&E) and TUNEL staining. Quantitative PCR (qPCR) and Western blotting were employed to measure the expression of relevant genes and the activation of signaling pathway proteins in lung tissues. Strain S_KLB exhibited robust growth on both MacConkey and SS agar media. It demonstrated resistance to a majority of tested antibiotics, including imipenem and oxacillin, and harbored a diverse virulence gene profile encompassing siderophores, fimbriae, efflux pumps, and metabolic factors. Animal challenge revealed that S_KLB infection induced severe pneumonia, characterized by significant weight loss, pulmonary edema, extensive neutrophil infiltration, and increased cellular apoptosis. At the molecular level, infected lung tissues showed marked upregulation of pro-inflammatory cytokines (*TNF-α, IL-6, IL-1β*) and the chemokine *CXCL1.* Concurrently, the NF-κB, STAT3, and p38 MAPK signaling pathways were activated, accompanied by elevated expression of the apoptotic protein Cleaved Caspase-3. The ground thrush-derived *K. aerogenes* strain S_KLB is a multidrug-resistant isolate possessing a broad range of virulence associated genes. It induces severe pneumonic injury by activating the NF-κB/STAT3/p38 MAPK signaling cascades, which drive excessive inflammatory responses and cellular apoptosis. This study provides experimental evidence for assessing the pathogenic potential of a wildlife-originating bacterial strain in a murine pneumonia model.

## Graphical Abstract

**Figure:**
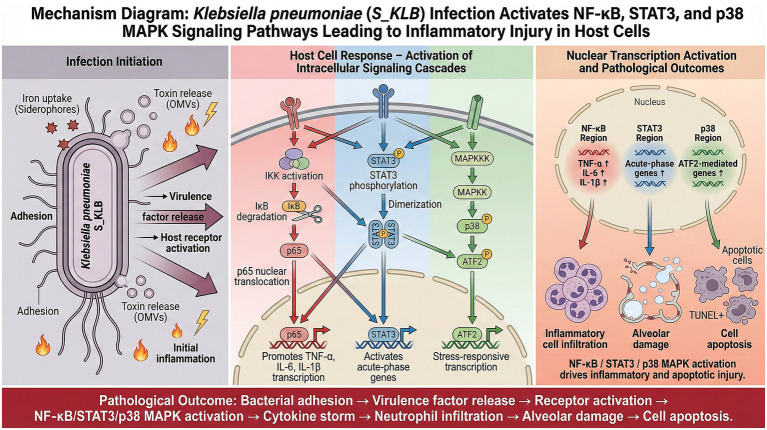

## 1 Introduction

A ubiquitous member of the *Enterobacteriaceae* family, *Klebsiella aerogenes* is recognized both as a common commensal and a clinically significant opportunistic pathogen. It is, for instance, frequently implicated in healthcare-associated infections, most notably pneumonia (Pan et al., 2021; Li et al., 2024). The public health threat it poses is significantly amplified by the ongoing global dissemination of multidrug-resistant strains (Morgado et al., 2024). Its pathogenesis is now understood to arise from complex and dynamic interactions between a suite of bacterial virulence determinants—including fimbriae, capsules, and various siderophore systems—and the host’s ensuing immune response (Abbas et al., 2024; Feng et al., 2024; Morgado et al., 2024).

The “One Health” paradigm has increasingly drawn attention to the role of wildlife as potential reservoirs for a range of pathogens. Migratory birds, given their extensive movements, are of particular interest in this context, as they may serve as vectors capable of disseminating antimicrobial-resistant and virulent bacterial strains across vast geographical boundaries (Mthembu et al., 2019; Napit et al., 2025). In veterinary contexts, *K. aerogenes* has been implicated in mastitis in dairy cattle, septicemia in poultry, and respiratory infections in companion animals (Lee et al., 2021; Li et al., 2021; Song et al., 2023; Martin et al., 2025). Notably, recent genomic surveillance studies have revealed that wildlife-origin *K. aerogenes* strains often harbor resistance genes and virulence determinants comparable to those found in clinical isolates, raising concerns about environmental reservoirs and cross-species transmission (Kutilova et al., 2018; Sun et al., 2024). Despite this recognition, systematic investigations specifically addressing the pathogenic potential of *K. aerogenes* strains originating from wild birds remain surprisingly scarce.

A considerable body of research has extensively characterized clinical isolates of *K. aerogenes*. Nevertheless, several critical knowledge gaps persist (Feng et al., 2024). First, strains derived from wildlife—particularly those from avian hosts—have received comparatively insufficient attention. Second, investigations have frequently centered on the role of isolated virulence factors. Consequently, they often fail to capture the complex, network-level interactions that occur between a strain’s putative virulence repertoire and the host’s multifaceted signaling responses. Third, there is a notable absence of studies employing standardized animal infection models, which are essential for a rigorous evaluation of the *in vivo* pathogenicity and immunopathological consequences of avian-origin strains. Finally, the extent of pathogenic heterogeneity among strains inhabiting distinct ecological niches, and its potential implications, remains poorly characterized. Collectively, these gaps impose significant limitations on developing a truly holistic understanding of *K. aerogenes* evolutionary dynamics and its potential for cross-species transmission.

It is in direct response to these very deficiencies that the present study focuses on a specific *K. aerogenes* strain, designated S_KLB, which was isolated from the lung tissue of a deceased ground thrush. Operating explicitly within a “One Health” framework, this study aims to systematically elucidate the infection mechanisms of this strain within the context of the mammalian respiratory system. The specific objectives are, therefore: (i) to perform accurate taxonomic identification and thorough biological characterization of strain S_KLB; (ii) to comprehensively profile its virulence gene repertoire and determine its antibiotic susceptibility pattern; (iii) to establish a murine pneumonia model for assessing its *in vivo* pathogenicity and associated histopathological alterations; (iv) to elucidate, at both the transcriptional and protein levels, the activation of key host signaling pathways (NF-κB, STAT3, p38 MAPK) and the expression of apoptosis-related molecules following infection (Zhang et al., 2026); and (v) to integrate these multi-level findings into a systematic model of S_KLB pathogenesis, thereby directly linking microbial genetic features with host molecular responses.

A review of the relevant literature confirms that type I and type III fimbriae are key mediators of adherence and biofilm formation, respectively (Birgani et al., 2025). Siderophore systems are understood to be crucial for bacterial iron acquisition in the host, while genes involved in capsule synthesis are known to influence anti-phagocytic capacity (Kong et al., 2021). Furthermore, efflux pump systems appear to play a dual role, contributing to both antimicrobial resistance and the regulation of virulence (Wan et al., 2022; Chenhaka et al., 2023). The host response to such infections is orchestrated by a network of signaling pathways, prominently including NF-κB (a master regulator of inflammation), STAT3 (involved in immune modulation and acute-phase responses), and p38 MAPK (linked to cellular stress and apoptosis) (Fan et al., 2013; Hoesel and Schmid, 2013; Wang et al., 2024). It is important to note, however, that existing studies—predominantly focused on clinical isolates—seldom provide a dynamic, *in vivo* correlation that successfully links a strain’s complete virulence arsenal with the concurrent activation of multiple host signaling pathways. It is precisely this critical gap that the present study is designed to address.

To achieve these objectives, a carefully considered, multi-tiered methodological approach was employed. At the bacterial level, strain identification was rigorously performed using a combination of classical microbiological culture, standard biochemical tests, and 16S rRNA gene sequencing. Biological characterization subsequently encompassed growth curve analysis, antibiotic susceptibility testing (employing the Kirby-Bauer disc diffusion method), and PCR-based detection targeting a panel of virulence genes. At the animal model level, an acute pneumonia model was successfully established through intranasal inoculation of mice. Disease severity was then assessed using a composite of parameters: clinical scoring, continuous body weight monitoring, gross pathological observation of lung tissue. Histopathological evaluation of inflammation and tissue injury was subsequently conducted using Hematoxylin and Eosin (H&E) staining. In parallel, cellular apoptosis within the lung tissue was detected and quantified via TUNEL staining (Mazzon and Cuzzocrea, 2007; Liu et al., 2013). For probing the underlying mechanisms, quantitative real-time PCR (qRT-PCR) was employed to precisely measure changes in the mRNA expression levels of genes implicated in inflammation, oxidative stress, and tissue remodeling (Chou et al., 2025). Complementing this, protein immunoblotting (Western blot) was utilized to analyze the phosphorylation states of key signaling proteins—specifically, NF-κB p65, STAT3, and p38 MAPK—and to assess the expression levels of the apoptotic effector protein, Cleaved Caspase-3 (Liu et al., 2025). This carefully constructed research design—progressing logically from *in vitro* characterization to *in vivo* validation—ensures a systematic and coherent elucidation of the pathogenic principles governing S_KLB infection.

## 2 Materials and methods

### 2.1 Bacterial isolation and molecular identification

The experimental strain was isolated from the lung tissue of a deceased ground thrush. The deceased ground thrush was collected in Tongliao City, Inner Mongolia, China (approximately 43°N, 122°E) on September 18, 2024. Ground thrushes are partial migrants; northern populations migrate south in autumn (September–October) to winter in southern China and Southeast Asia. Tongliao is located along the flyway, a major stopover site for migratory birds, with hundreds of thousands of birds passing through during migration seasons. Under aseptic conditions, lung tissue samples were homogenized in sterile phosphate-buffered saline (PBS), plated onto Luria-Bertani (LB) agar, and incubated aerobically at 37 °C for 18–24 h, following the protocol described by Müller et al. (2024). Predominant colonies were subsequently sub-cultured via streak plating on fresh LB agar to obtain pure, morphologically uniform isolates.

For phenotypic characterization, purified colonies were streaked onto selective and differential media, including Salmonella-Shigella (SS) agar, MacConkey agar, and Columbia blood agar, then incubated under identical conditions. Gram staining was subsequently performed, and cellular morphology was examined using light microscopy.

Genomic DNA was extracted using the Tiangen Bacterial Genomic DNA Extraction Kit (DP302). The 16S rRNA gene was amplified via polymerase chain reaction (PCR) using the universal primer pair 27F and 1492R. Each 50 μL reaction mixture contained 25 μL of 2 × Taq Plus Master Mix, 2 μL of each primer (10 μM), 2 μL of DNA template, and nuclease-free water to volume. The thermal cycling conditions were as follows: initial denaturation at 94 °C for 5 min; 35 cycles of denaturation at 94 °C for 30 s, annealing at 55 °C for 30 s, and extension at 72 °C for 90 s; followed by a final extension at 72 °C for 7 min. PCR products were resolved by electrophoresis on a 1% agarose gel, purified, and subsequently submitted to Sangon Biotech for bidirectional Sanger sequencing.

The resulting sequencing chromatograms were assembled and analyzed using SnapGene software. The consensus sequence was then compared against the NCBI nucleotide database using BLAST. A phylogenetic tree was subsequently constructed in MEGA 11 software employing the neighbor-joining method with 1,000 bootstrap replicates, as described by Gibbon et al. (2026). The tree included 16S rRNA sequences from reference strains of *Klebsiella aerogenes* (MK368411.1, PP702223.1, NR 114737.1, NR 102493.2), *Klebsiella electrica* (NR 125461.1)*, Klebsiella terrigena* (NR 037085.1), *Klebsiella pneumoniae* (NR 114506.1), *Klebsiella* var*iicola* (NR 025635.1), *Klebsiela afncana* (NR 180233.1), *Klebsiella quasipneumoniae* (NR 134062.1), *Klebsiela michiganensis* (NR 118335.1), *Klebsiella grimonti* (NR 159317.1), and *Escherichia coli* (NR 024570.1, NR 114042.1) as an outgroup, retrieved from the GenBank database. Based on this integrated analysis, the isolate was designated strain S_KLB.

### 2.2 Growth kinetics analysis

The growth kinetics of strain S_KLB were determined using the following procedure. First, the cryopreserved stock of the isolate was subjected to two successive rounds of activation on appropriate medium. Subsequently, an inoculum of 2% (v/v) was transferred into 200 mL each of fresh LB broth and Brain Heart Infusion (BHI) broth, contained within separate 250 mL conical flasks. Cultures were then incubated at 37 °C under constant agitation at 160 rpm in a shaking incubator, following the method described by Pollack-Milgate et al. (2024). Commencing immediately after inoculation, 5 mL aliquots of each culture were aseptically collected at two-hour intervals. For each time point, the optical density at 600 nm (OD600) was measured immediately using a spectrophotometer, with sterile broth serving as the blank reference. Concurrently, a 100 μL sample from each aliquot was serially diluted tenfold in sterile saline, according to the protocol of Pollack-Milgate et al. (2024). From two to three appropriate dilution levels, 100 μL aliquots were spread onto duplicate agar plates and incubated at 37 °C for 48 h to allow for colony enumeration. A comprehensive growth curve was subsequently constructed for each culture medium by plotting incubation time on the abscissa against both OD600 values and the logarithmic values of viable bacterial counts (log₁₀ CFU/mL) on the ordinate. This analysis enabled the precise determination of key growth phases, including the lag phase, the logarithmic (exponential) phase, and the stationary phase (Pollack-Milgate et al., 2024).

### 2.3 Antimicrobial susceptibility testing

Antimicrobial susceptibility of strain S_KLB was assessed using the standardized Kirby-Bauer disk diffusion method. An overnight bacterial culture was first adjusted with sterile physiological saline to a turbidity equivalent to the 0.5 McFarland standard, corresponding to approximately 1–2 × 10^8^ CFU/mL, following the procedure described. A sterile cotton swab was then saturated with the adjusted suspension and rotated against the inner wall of the tube to remove excess fluid. This swab was subsequently used to inoculate the entire surface of a Mueller-Hinton agar plate. To ensure uniform distribution, the inoculum was spread evenly by streaking the swab in three directions, with the plate rotated approximately 60° between each streaking. The inoculated plates were then allowed to dry at room temperature for 5 min, as recommended. Thereafter, antibiotic-impregnated disks were aseptically placed onto the agar surface using sterile forceps. Care was taken to maintain a center-to-center distance of at least 24 mm between adjacent disks. After a 15-min incubation period at room temperature in an upright position to allow for initial diffusion, the plates were inverted and incubated at 37 °C for 18–24 h. Following incubation, the diameters of the complete zones of inhibition, including the disk diameter, were measured on the reverse side of each plate using a calibrated Vernier caliper with an accuracy of 0.1 mm, as described by Matuschek et al. (2014). *Escherichia coli* ATCC 25922 and *Staphylococcus aureus* ATCC 25923 were used as quality control strains, and all tests were performed in duplicate to ensure reproducibility. Interpretation of the results—categorizing the strain as susceptible (S), intermediate (I), or resistant (R)—was performed in strict accordance with the species-specific clinical breakpoints for *Klebsiella aerogenes* as defined by the Clinical and Laboratory Standards Institute (CLSI). For agents lacking specific *K. aerogenes* breakpoints, interpretive criteria for *Enterobacteriaceae* were used. However, validated CLSI breakpoints for *K. aerogenes* are not available for oxacillin, macrolides, teicoplanin, rifampicin, and polymyxin B; results for these agents are presented for completeness and should be interpreted with caution, as they were not used as primary evidence for multidrug-resistance classification. These breakpoints were derived from the latest editions of the Clinical and Laboratory Standards Institute (CLSI) documents, specifically M100 (33rd edition) and VET08, as cited by Schuetz et al. (2025).

### 2.4 Virulence gene profiling via PCR

To assess the pathogenic potential of *Klebsiella aerogenes* strain S_KLB, a PCR-based screening approach was employed to detect the presence of 13 established virulence-associated genes, following the panel described by Li et al. (2026). The selected gene set encompassed several functional categories: genes involved in siderophore biosynthesis (*entB, iroB, iroN*); those encoding fimbrial adhesins (*fimA, mrkA, mrkB*); regulators of capsular synthesis (*wecB, rcsA, rcsB*); components of efflux pump systems (*AcrA, AcrB*); and metabolism-associated virulence determinants (*astA, astD*). Primer sequences for these targets were retrieved from the GenBank database and are listed in Supplementary Table 1. Representative PCR products were verified by sequencing to ensure target accuracy (data not shown). Genomic DNA extracted from strain S_KLB served as the template for individual PCR amplifications. Each 25 μL reaction mixture contained the following components: 12.5 μL of 2 × Taq PCR Master Mix, 0.5 μL of each forward and reverse primer (10 μM), 2 μL of DNA template, and 9.5 μL of nuclease-free ddH₂O, following the protocols of Weehuizen et al. (2016) and Birgani et al. (2025). For each PCR reaction, a no template control (NTC) (nuclease-free water in place of template DNA) was included to exclude contamination. Positive controls were not included due to the unavailability of a reference strain for each target gene. To ensure amplification specificity, representative PCR products from each target were verified by sequencing, confirming the correct amplification of the intended gene fragments (data not shown). All reactions were performed in duplicate to ensure reproducibility. Thermal cycling conditions were optimized for each primer pair; a representative general program consisted of an initial denaturation at 95 °C for 5 min, followed by 30 cycles of denaturation at 95 °C for 30 s, annealing at 55 °C for 30 s, and extension at 72 °C (with extension time adjusted according to the expected amplicon size), as described by Zhang et al. (2026). A final extension step was performed at 72 °C for 7 min. Following amplification, 5 μL aliquots of each PCR product were resolved by electrophoresis on a 1.5% agarose gel stained with ethidium bromide. DNA bands were visualized under ultraviolet (UV) illumination, and the presence of each target gene was determined by comparing the observed band sizes against a standard DNA molecular weight marker.

### 2.5 Experimental animals

A total of twelve 6-week-old male Kunming mice, of specific pathogen-free (SPF) grade, were procured from Beijing Huafukang Biotechnology Co., Ltd. for use in this study. Upon arrival, all animals were acclimatized for 7 days within an individually ventilated caging system, which was maintained under controlled environmental conditions. These included a constant temperature of 22 ± 2 °C, a relative humidity of 55% ± 10%, and a 12-h light/dark cycle, as described by Mo et al. (2025). During this acclimatization period, the mice had ad libitum access to irradiated standard laboratory rodent chow and autoclaved drinking water. All animal experimental procedures were conducted in strict compliance with the Guidelines for the Ethical Review of Laboratory Animal Welfare in China and were formally approved by the Institutional Animal Care and Use Committee. The experimental design employed a randomized grouping strategy, with predefined humane endpoints established prior to the initiation of the study.

### 2.6 Establishment of a murine pulmonary infection model

Following the acclimatization period, the 12 mice were randomly assigned to either an experimental infection group (*n* = 6) or a control group (*n* = 6) using a random number table, following the method described by Fann et al. (2018). Pulmonary infection was subsequently established via intranasal instillation. Prior to the procedure, mice were fasted for 4 h while maintaining free access to water, as outlined by Kumar and Chhibber (2011). Deep anesthesia was induced via isoflurane inhalation, and its adequacy was confirmed by the absence of a pedal reflex upon hindlimb pinch. Once anesthetized, mice were positioned in dorsal recumbency with the head held upright. A 50 μL aliquot of the prepared inoculum was then administered slowly and dropwise to a single nostril using a micropipette. This technique allowed the animal to aspirate the liquid into the lower respiratory tract through spontaneous inhalation. The experimental group received a suspension of *Klebsiella aerogenes* strain S_KLB in sterile phosphate-buffered saline (PBS) at a concentration of 1 × 10^8^ CFU/mL, while the control group received an equal volume of sterile PBS alone. Throughout the instillation procedure, respiratory patterns were closely monitored to ensure complete aspiration. Following inoculation, mice were placed on a warming pad in lateral recumbency until they had fully recovered from anesthesia, after which they were returned to their home cages. Animals were subsequently monitored continuously for clinical signs, including changes in activity levels, food and water intake, fur condition, and respiratory patterns. At 24 h post-infection, all mice were humanely euthanized via cervical dislocation. Lung tissues were harvested immediately for subsequent analyses, which included, histopathological examination, and assessment of inflammatory cytokine levels.

### 2.7 Histopathological analysis of lung tissues

To evaluate pulmonary damage and cellular apoptosis following infection, lung tissue samples collected 24 h post-challenge were examined using hematoxylin and eosin (H&E) staining and the terminal deoxynucleotidyl transferase dUTP nick end labeling (TUNEL) assay. Tissue blocks from the left inferior lobe of each mouse were fixed in 4% paraformaldehyde for 24 h, following the protocol described by Lee et al. (2024). After fixation, the tissues were paraffin-embedded and subsequently sectioned at a thickness of 4–5 μm. For H&E staining, sections were deparaffinized, rehydrated, and stained according to standard histological protocols. Pathological changes—including alterations in alveolar integrity, the extent of inflammatory infiltration, and the presence of edema and hemorrhage—were then assessed under light microscopy, as outlined by Mei et al. (2019) and Schniering et al. (2018). Apoptosis was detected using a one-step TUNEL apoptosis detection kit, strictly following the manufacturer’s instructions. Briefly, after deparaffinization, rehydration, and permeabilization with Proteinase K, the sections were incubated with the TUNEL reaction mixture at 37 °C for 60 min in a humidified, light-protected chamber, according to the method of Luo et al. (2025). Following washes with phosphate-buffered saline (PBS), nuclei were counterstained with DAPI, and the slides were mounted with an anti-fade mounting medium, as described by Schniering et al. (2018).

### 2.8 Quantitative real-time PCR analysis of gene expression

To evaluate immune, oxidative stress, and tissue remodeling responses in murine lung tissue following *Klebsiella aerogenes* infection, the mRNA expression levels of eight key genes were quantified using quantitative reverse transcription PCR (qRT-PCR), following the approach described by Mark et al. (2014). The selected gene panel was chosen based on their established roles in host responses to Gram-negative bacterial pneumonia. Pro-inflammatory cytokines (*Tnfα*, *Il6*, *Il1b*) and the chemokine *Cxcl1* were selected as key mediators of neutrophil recruitment and inflammatory amplification (Jones et al., 2006; José et al., 2015; Xiao et al., 2025). *Hamp* (hepcidin) was included to assess nutritional immunity responses, as iron restriction is a critical host defense mechanism against bacterial infection (Stefanova et al., 2017; Nairz et al., 2018). *Nrf2* and *Hmox1* were selected to evaluate oxidative stress responses, while Mmp9 was included as a marker of tissue remodeling and potential alveolar damage (José et al., 2015; Gomez et al., 2016; Romero-Durán et al., 2024). The selected gene panel encompassed several functional categories: pro-inflammatory cytokines (*Tnfα, Il6, Il1b*); a chemokine (*Cxcl1*); an iron metabolism regulator (*Hamp*); oxidative stress regulators (*Nrf2, Hmox1*); and a matrix metalloproteinase (*Mmp9*). The primer sequences used for qPCR are listed in Supplementary Table 2. Total RNA was extracted from approximately 30 mg of lung tissue harvested 24 h post-infection using TRIzol reagent, according to the manufacturer’s protocol. RNA concentration and purity were assessed spectrophotometrically, with an acceptable A260/A280 ratio falling between 1.8 and 2.0, as specified by Alimu et al. (2025). Subsequently, one microgram of RNA was reverse-transcribed into complementary DNA (cDNA) using PrimeScript™ RT Master Mix. Quantitative real-time PCR was then performed with SYBR Green detection, employing gene-specific primers that were either designed using Primer-BLAST or obtained from published literature. The housekeeping gene *GAPDH* served as the internal reference, following the method of Seo et al. (2010). Each 20 μL reaction mixture contained the following components: 10 μL of 2 × SYBR Green Pro Taq HS Premix, 0.8 μL of each forward and reverse primer (10 μM), 2 μL of cDNA template, and nuclease-free water to volume. The thermal cycling conditions were as follows: initial denaturation at 95 °C for 30 s; 40 cycles of denaturation at 95 °C for 5 s and annealing/extension at 60 °C for 30 s; followed by a melt curve analysis to verify amplification specificity, as described by Wang et al. (2019). All samples were run in six technical replicates to ensure reproducibility. Relative gene expression levels were calculated using the 2^−ΔΔCt^ method, with *GAPDH* serving as the normalizing reference. The average of the six technical replicates was used as a single data point for statistical analysis, with each mouse considered an independent biological replicate (n = 6 per group).

### 2.9 Western blot analysis

To assess the activation status of key signaling pathways in lung tissue following *Klebsiella aerogenes* infection, Western blot analysis was performed, following the approach described by Fan et al. (2020). The selected signaling proteins—NF-κB p65, STAT3, and p38 MAPK—were chosen based on their central roles in orchestrating inflammatory responses during bacterial pneumonia. NF-κB is a master regulator of pro-inflammatory cytokine transcription, STAT3 mediates acute-phase responses and immune modulation, and p38 MAPK is critically involved in stress responses, cytokine production, and apoptosis (Fan et al., 2013; Hoesel and Schmid, 2013; Wang et al., 2024). Cleaved Caspase-3 was assessed as an executioner of apoptosis to evaluate tissue injury mechanisms (Duan et al., 2003). The targets examined included key components of the NF-κB, STAT3, and p38 MAPK pathways, along with an apoptotic effector protein. Specifically, the phosphorylated and total forms of NF-κB p65, STAT3, and p38 MAPK were evaluated, as well as cleaved Caspase-3, according to the panel used by Feng et al. (2019). Lung tissue samples were homogenized in ice-cold RIPA lysis buffer supplemented with a cocktail of protease and phosphatase inhibitors, following the protocol of Lee et al. (2024). After a 30-min incubation on ice, the homogenates were centrifuged at 12,000 × g for 15 min at 4 °C, and the resulting supernatants were collected. Protein concentration in each sample was determined using a bicinchoninic acid (BCA) assay. Equal amounts of protein (30 μg per lane) were then mixed with 5× Laemmli buffer, denatured at 100 °C for 5 min, and separated by sodium dodecyl sulfate-polyacrylamide gel electrophoresis (SDS-PAGE) on 10% or 12% gels, as described by Sharata et al. (2025) and Mankonkwana et al. (2025). Following electrophoresis, the resolved proteins were transferred onto polyvinylidene fluoride (PVDF) membranes. The membranes were subsequently blocked with 5% non-fat milk in Tris-buffered saline with Tween 20 (TBST) for 1 h at room temperature to prevent non-specific binding. This was followed by overnight incubation at 4 °C with primary antibodies targeting the following proteins: phosphorylated NF-κB p65 (p-NF-κB p65, 1:1000 dilution), total NF-κB p65 (1:1000), phosphorylated STAT3 (p-STAT3, 1:1000), total STAT3 (1:1000), phosphorylated p38 MAPK (p-p38 MAPK, 1:1000), total p38 MAPK (1:1000), cleaved Caspase-3 (1:500), and the housekeeping protein GAPDH (1:2000), following the dilutions recommended by Mani and Arfeen (2024). After extensive washing with TBST, the membranes were incubated with horseradish peroxidase (HRP)-conjugated secondary antibodies (1:4000 dilution) for 1 h at room temperature. Protein bands were visualized using an enhanced chemiluminescence (ECL) substrate and detected with a digital imaging system. Band intensities were subsequently quantified using ImageJ software. Target protein expression levels were normalized to the GAPDH loading control, as described by Alonso Villela et al. (2020), and phosphorylation levels were expressed as the ratio of phosphorylated protein to total protein for each respective target. Densitometric quantification was performed from at least three independent experiments, and statistical comparisons were conducted on the normalized values.

### 2.10 Statistical analysis

All statistical analyses were performed using Origin 2024 (OriginLab Corporation, Northampton, MA, USA). Data are presented as mean ± standard deviation (SD) from at least three independent biological replicates, unless otherwise specified. For the animal experiments, six mice per group were used, and each mouse was considered an independent biological replicate.

Comparisons between two groups were analyzed using unpaired two-tailed Student’s *t*-test. For comparisons involving more than two groups or repeated measurements (e.g., body weight changes over time), two-way analysis of variance (ANOVA) followed by Bonferroni’s or Sidak’s post-hoc test was applied. Normality of data distribution was assessed using the Shapiro–Wilk test, and homogeneity of variances was verified using Levene’s test prior to parametric analysis. For data that did not meet normality assumptions, non-parametric alternatives (Mann–Whitney U test) were used.

For qPCR analysis, each sample was run in six technical replicates to ensure measurement precision; the average of these technical replicates was calculated and treated as a single data point for statistical analysis. Biological replicates consisted of independent RNA extractions from different mice, with six mice per group.

For Western blot analysis, densitometric quantification was performed using ImageJ software. Protein expression levels were normalized to GAPDH as a loading control, and phosphorylation levels were expressed as the ratio of phosphorylated protein to total protein. Statistical comparisons were performed on normalized values from at least three independent experiments.

A significance threshold of *p < 0.05 was used for all analyses, with significance levels denoted as *p < 0.05, **p < 0.01, and ***p < 0.001. No additional multiple-comparison correction was applied beyond the post-hoc tests described above, as the analyses were hypothesis-driven with pre-specified comparisons.

## 3 Results

### 3.1 Isolation and identification of strain S_KLB

**Figure 1:**
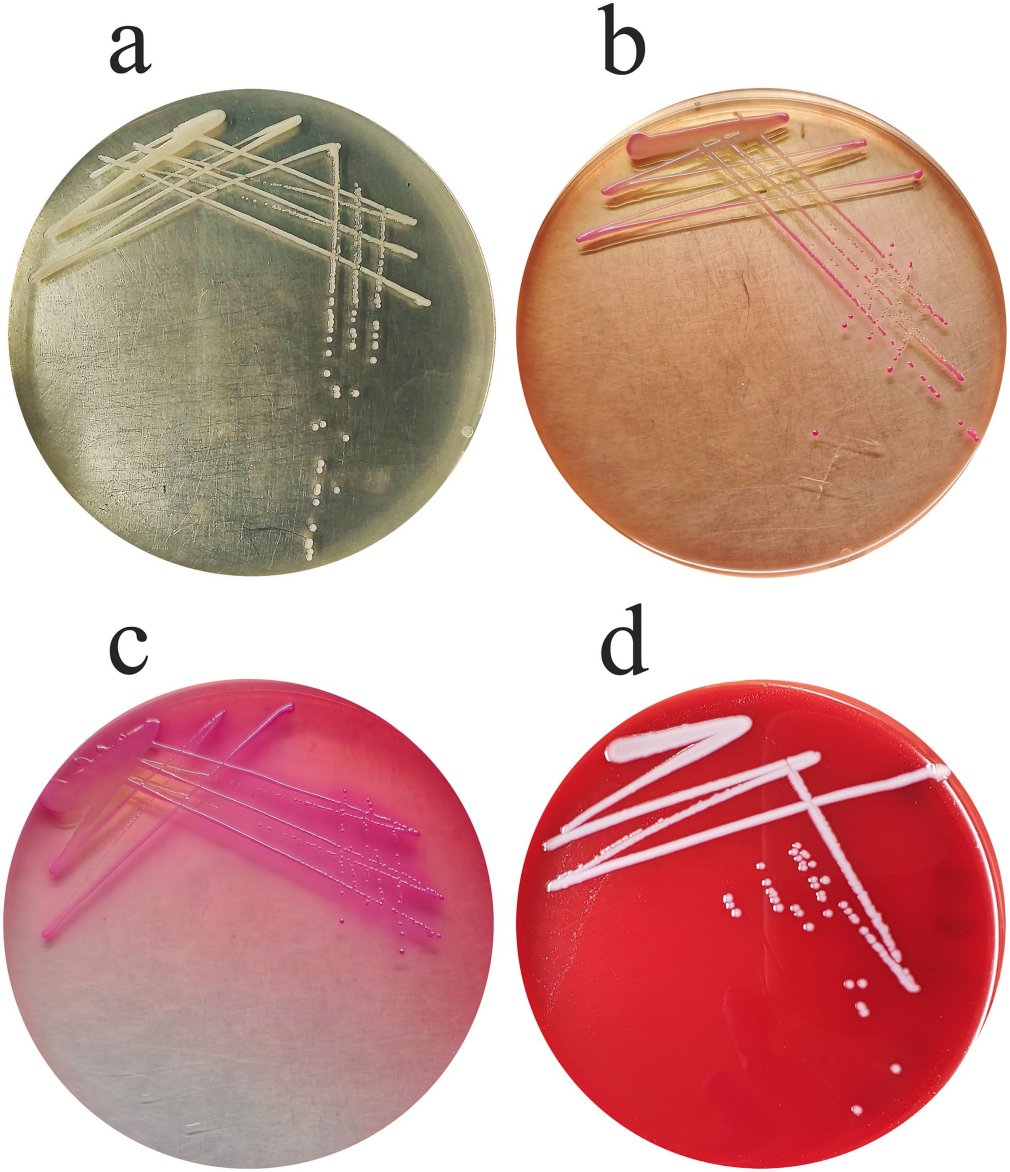
Morphological characterization of *Klebsiella aerogenes* strain S_KLB on various culture media. **(a)** Colonies of strain S_KLB on LB agar plate, showing smooth, moist, mucoid, and greyish-white morphology. **(b)** Growth of strain S_KLB on MacConkey agar, displaying characteristic pink colonies indicating lactose fermentation. **(c)** Growth of strain S_KLB on SS agar, with colonies resembling those on MacConkey agar without growth inhibition. **(d)** Colonies of strain S_KLB on Columbia blood agar plate, appearing white, smooth, and moist with non-hemolytic (*γ*-hemolytic) zones.

The bacterial strain isolated from the lung tissue of the deceased ground thrush formed smooth, moist, mucoid, and greyish-white colonies on Luria-Bertani (LB) agar plates (Figure 1a). Following purification, the strain was cultured on selective and differential media, including MacConkey agar and Salmonella-Shigella (SS) agar (Figures 1b,c). It exhibited robust growth on both media, with no significant differences observed in colony morphology, size, or density. On MacConkey agar, colonies displayed a characteristic pink coloration, thereby confirming their capability to ferment lactose. On SS agar, colony morphology resembled that observed on MacConkey agar, with no detectable complete growth inhibition. When cultured on Columbia blood agar plates, colonies appeared white, smooth, and moist, measuring approximately 2–3 mm in diameter, and were surrounded by a non-hemolytic (gamma-hemolytic) zone (Figure 1d).

**Figure 2:**
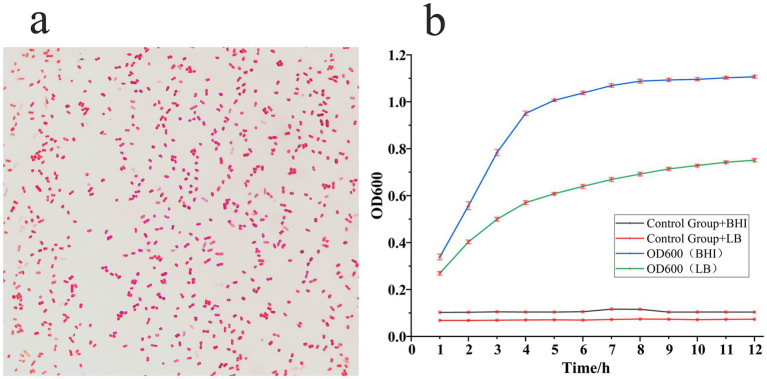
Morphological and growth kinetic analysis of strain S_KLB. **(a)** Gram staining of strain S_KLB, showing Gram-negative short rod-shaped bacteria (scale bar: 5 μm). **(b)** Growth curves of strain S_KLB cultured in different media: Blue curve: Strain S_KLB in BHI broth, Green curve: Strain S_KLB in LB broth, Black curve: Sterile BHI broth (negative control), Red curve: Sterile LB broth (negative control), the optical density at 600 nm (OD600) was monitored over time.

Gram staining revealed the isolate to be a Gram-negative, short rod-shaped bacterium (Figure 2a). To characterize the *in vitro* growth kinetics of strain S_KLB, growth curves were subsequently determined in both LB and Brain Heart Infusion (BHI) liquid media. The results, presented in Figure 2b, indicated a short lag phase of less than 30 min in both media, followed by a rapid transition into the exponential growth phase. In LB broth, the strain entered the stationary phase approximately 6 h post-inoculation, reaching a maximum OD600 of about 0.8. In the nutrient-richer BHI medium, a steeper exponential phase slope was observed, with the stationary phase occurring earlier at around 4 h and a higher maximum OD600 of approximately 1.1. Viable colony counts obtained via plate enumeration closely mirrored the OD600 growth trends, further validating the typical growth pattern of the strain under these conditions.

**Figure 3:**
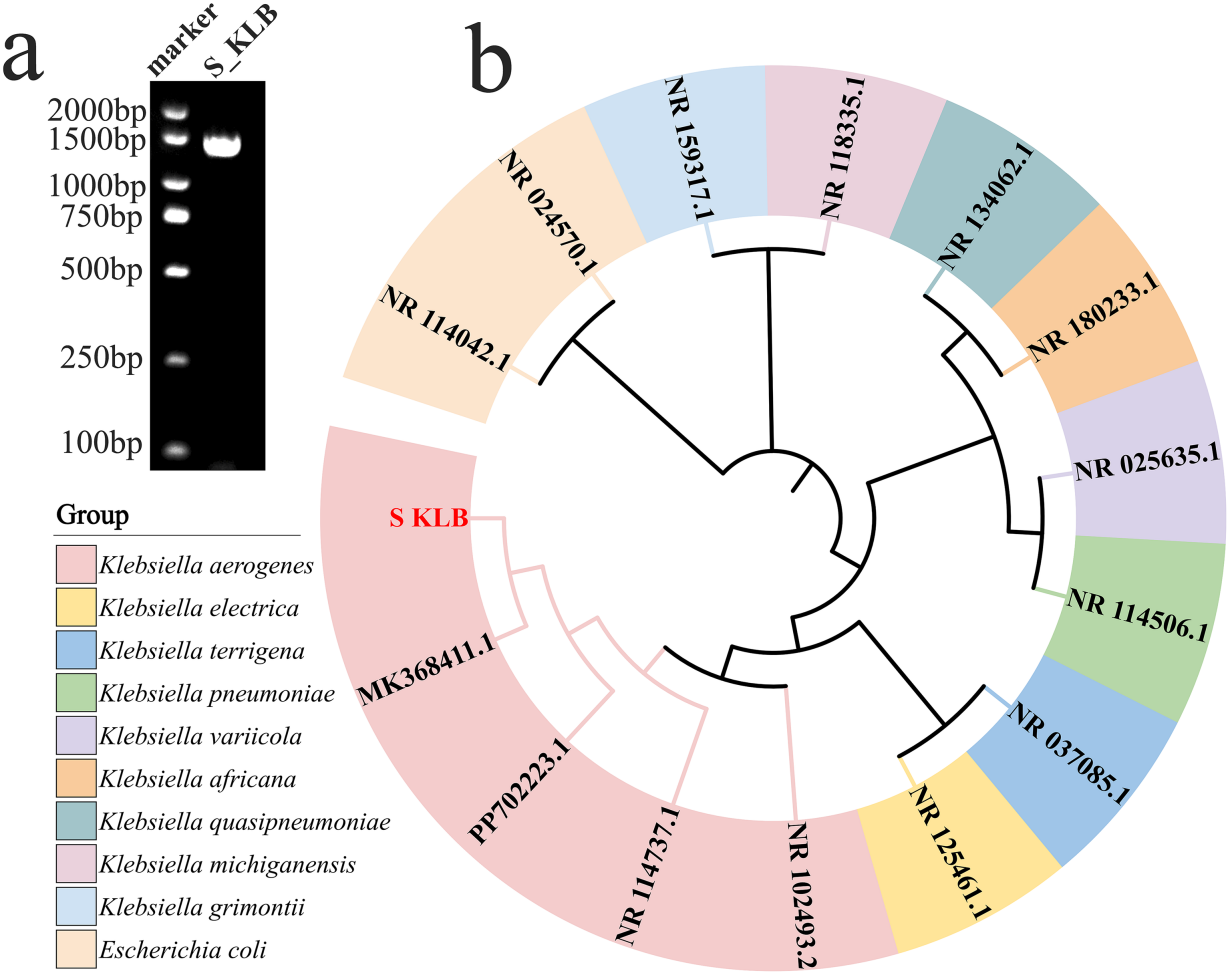
Molecular identification and phylogenetic analysis of strain S_KLB. **(a)** Agarose gel electrophoresis of PCR-amplified 16S rRNA gene from strain S_KLB, showing a single specific band of approximately 1,500 bp. Lane Marker: DNA molecular weight marker; Lane S_KLB: 16S rRNA PCR product. **(b)** Phylogenetic tree based on 16S rRNA gene sequences, showing the clustering of strain S_KLB (marked with a solid circle) within the *Klebsiella aerogenes* clade.

Polymerase chain reaction (PCR) amplification of the 16S rRNA gene from the extracted genomic DNA yielded a single, specific amplicon of approximately 1,500 base pairs, as shown in Figure 3a, consistent with findings reported by Fahim et al. (2025) and Sun et al. (2025). Sequencing and subsequent bioinformatic analysis revealed that this sequence shared 99.8% identity with reference *Klebsiella aerogenes* strains deposited in the GenBank database. Phylogenetic tree analysis further confirmed that the isolate S_KLB clustered within the same clade as the *K. aerogenes* type strain, with high bootstrap support, following the approach of Li et al. (2024) (Figure 3b). Collectively, these results—encompassing colony morphology, staining characteristics, biochemical reactions, and molecular identification—identified the isolate as *Klebsiella aerogenes*, which was subsequently designated strain S_KLB.

### 3.2 Antimicrobial resistance and virulence gene profiles of strain S_KLB

**Figure 4:**
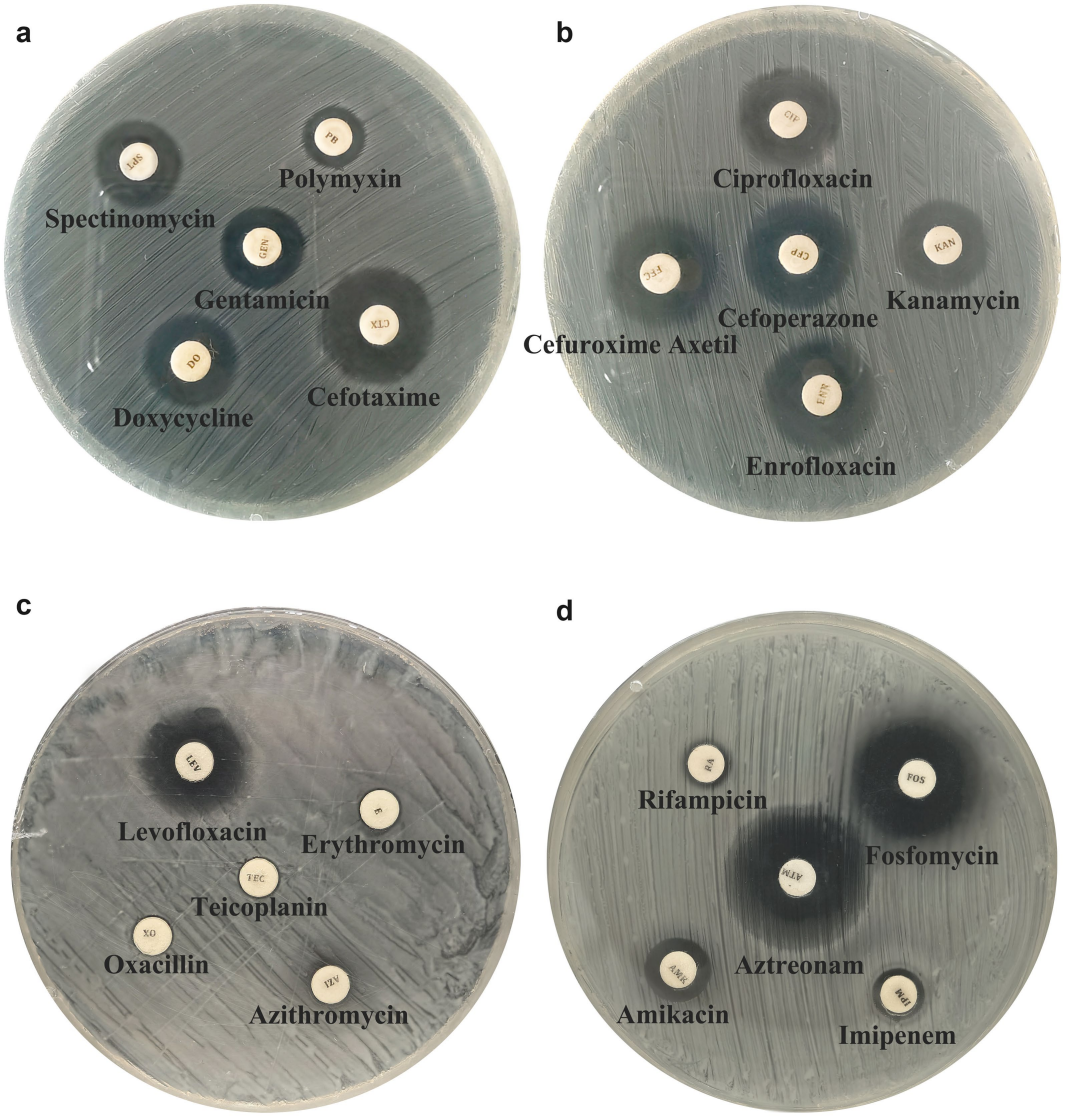
Antimicrobial susceptibility profile of *Klebsiella aerogenes* strain S_KLB. Disk diffusion assay results showing zones of inhibition (measured in millimeters) for strain S_KLB against 20 different antibiotics. Antibiotics are grouped by class: β-lactams, macrolides, aminoglycosides, cephalosporins, fluoroquinolones, and others. The dashed horizontal lines indicate clinical breakpoints for susceptibility (S), intermediate (I), and resistance (R) according to CLSI guidelines. Note the absence of inhibition zones for oxacillin, macrolides, and teicoplanin.

Antimicrobial susceptibility testing revealed that strain S_KLB exhibited a broad resistance profile against the panel of 20 antibiotics tested (Table 1; Figure 4). Among the *β*-lactams, S_KLB was resistant to imipenem, with an inhibition zone diameter of 9 mm (Table 1). For oxacillin, macrolides, teicoplanin, rifampicin, and polymyxin B, validated CLSI breakpoints for *K. aerogenes* are not available; these results are presented for completeness and should be interpreted with caution. All tested macrolides, including azithromycin and erythromycin, proved ineffective, yielding no zone of inhibition. For the aminoglycosides, the strain displayed intermediate susceptibility to gentamicin (14 mm) and kanamycin (15 mm) but was resistant to amikacin (11 mm). Among the cephalosporins, only cefoperazone (18 mm) showed intermediate susceptibility, while resistance was observed against cefotaxime (18 mm) and cefuroxime axetil (16 mm). Notably, S_KLB remained susceptible to a limited number of agents, including enrofloxacin (19 mm), fosfomycin (20 mm), and aztreonam (24 mm). However, resistance was noted against other fluoroquinolones such as ciprofloxacin (14 mm), and intermediate susceptibility was observed for levofloxacin (19 mm). Furthermore, the strain exhibited resistance to spectinomycin (14 mm), doxycycline (15 mm), teicoplanin (no zone), rifampicin (8 mm), and polymyxin B (10 mm). In summary, strain S_KLB displayed extensive multidrug resistance, particularly to β-lactams, macrolides, and select aminoglycosides and fluoroquinolones, while retaining susceptibility to only a limited number of antimicrobial agents.

**Table 1:** Detailed antimicrobial susceptibility testing results for strain S_KLB.

Drug class	Drug	Concentration	Bacteriostatic ring diameter (mm)	Result	Standard *f* criterion
Beta-lactams	Imipenem	10 μg	9	R	23(S), 20-22(I), 19(R)
	Oxacillin	10 μg	6	R	13(S), 11-12(I), 10(R)
Macrolides	Azithromycin	15 μg	6	R	12(S), 11(I), 10(R)
	Erythromycin	15 μg	6	R	23(S), 14-22(I), 13(R)
Aminoglycosides	Gentamicin	10 μg	14	I	15(S), 13-14(I), 12(R)
	Kanamycin	30 μg	15	I	18(S), 14-17(I), 13(R)
	Amikacin	30 μg	11	R	17(S), 15-16(I), 14(R)
Cephalosporins	Cefotaxime	30 μg	18	R	26(S), 23-25(I), 22(R)
	Cefoperazone	75 μg	18	I	21(S), 16-20(I), 15(R)
	Cefuroxime Axetil	30 μg	16	I	18(S), 15-17(I), 14(R)
Quinolones	Enrofloxacin	5 μg	19	S	19(S), 14-18(I), 13(R)
	Levofloxacin	5 μg	19	I	21(S), 17-20(I), 16(R)
Fluoroquinolones	Ciprofloxacin	10 μg	14	R	26(S), 22-25(I), 21(R)
Aminocyclitols	Spectinomycin	100 μg	14	R	18(S), 15-17(I), 14(R)
Tetracyclines	Doxycycline	30 μg	15	I	18(S), 15-17(I), 14(R)
Glycopeptides	Teicoplanin	30 μg	6	R	14(S), 11-13(I), 10(R)
Nitrofurans	Fosfomycin	200 μg	20	S	16(S), 13-15(I), 12(R)
Monobactams	Aztreonam	30 μg	24	S	21(S), 18–20(I), 17(R)
Ansamycins	Rifampicin	5 μg	8	R	20(S), 17-19(I), 16(R)
Polypeptides	Polymyxin	300 μg	10	R	≥14 (S), <14 (R)

**Figure 5:**
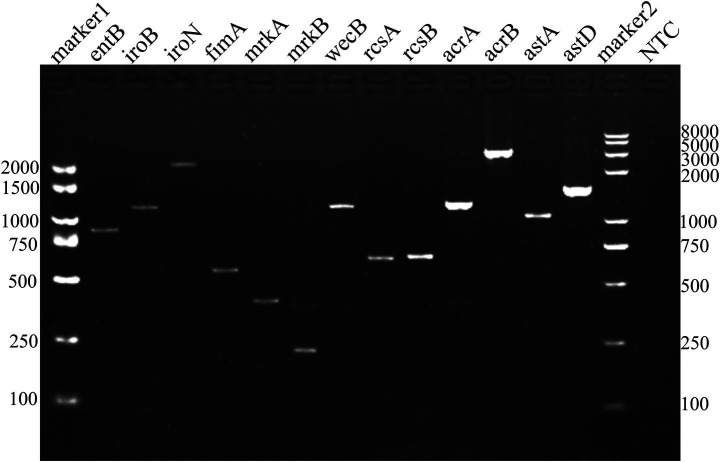
Virulence gene profile of *Klebsiella aerogenes* strain S_KLB. Agarose gel electrophoresis showing PCR amplification products of 13 virulence-associated genes from strain S_KLB. Lane Marker: DNA molecular weight marker. Lanes 1–14 correspond to: entB (1), iroB (2), iroN (3), fimA (4), mrkA (5), mrkB (6), wecB (7), rcsA (8), rcsB (9), AcrA (10), AcrB (11), astA (12), astD (13), no template control (NTC) (14). All lanes show specific amplification products at expected sizes.

PCR-based screening for 13 established virulence-associated genes demonstrated that strain S_KLB harbors a diverse virulence gene repertoire (Figure 5). The validation of PCR results using negative controls and sequencing confirmation is detailed in the Methods section (Section 2.4). All targeted genes were successfully amplified, including those involved in siderophore biosynthesis (*entB, iroB, iroN*); fimbrial adhesin-related functions (*fimA, mrkA, mrkB, wecB, rcsA, rcsB*); efflux pump systems (*AcrA, AcrB*); and metabolism-associated virulence determinants (*astA, astD*). This profile indicates that S_KLB possesses genes associated with multiple potential virulence mechanisms encompassing iron acquisition, adherence and colonization, efflux-mediated detoxification, and metabolic aggression, thereby suggesting possible pathogenic potential.

### 3.3 Impact of S_KLB infection on pulmonary inflammation and histopathology in mice

**Figure 6:**
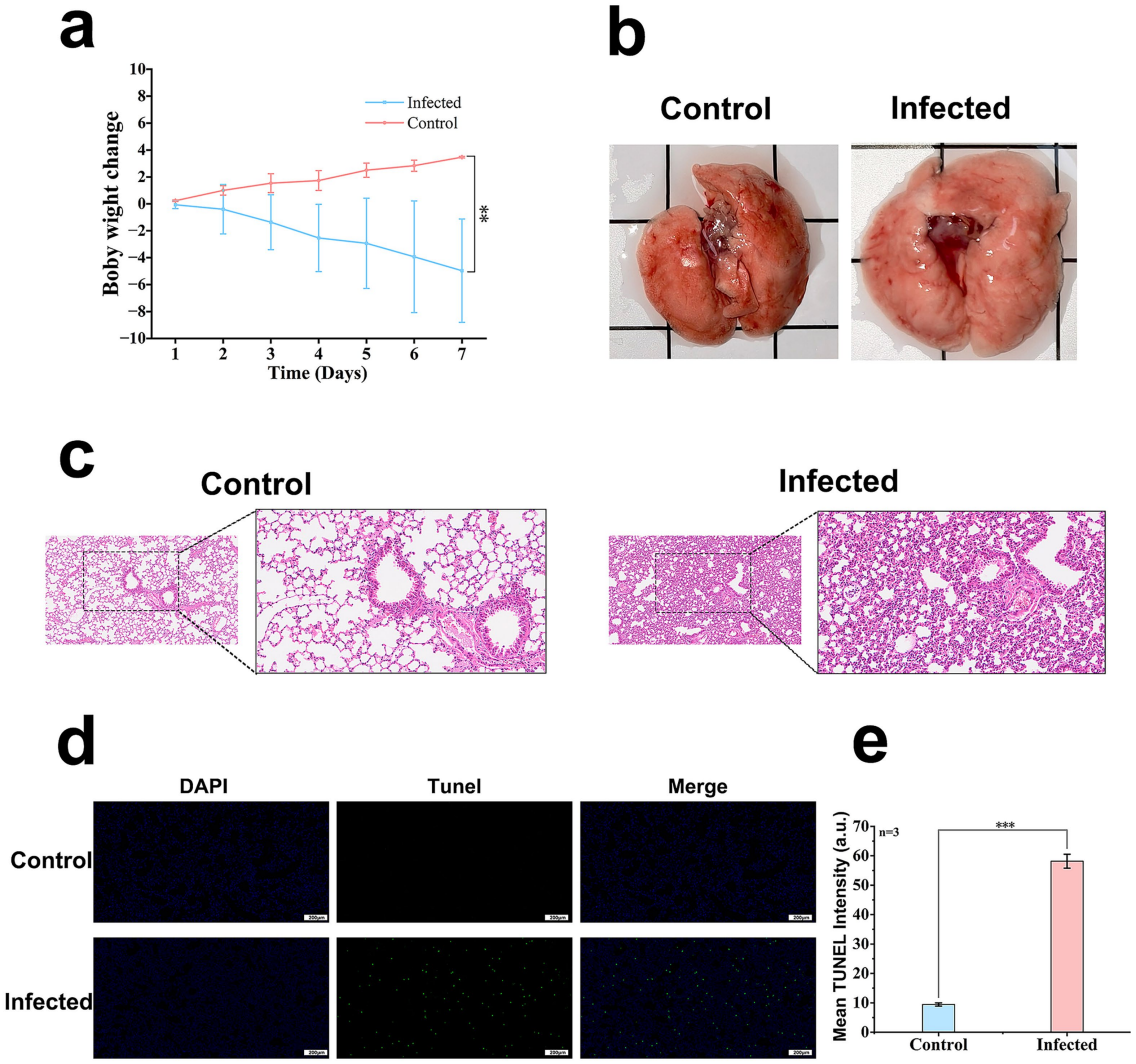
Pathological effects of *Klebsiella aerogenes* S_KLB infection in a murine pneumonia model. **(a)** Body weight changes in mice following intranasal infection with strain S_KLB (*n* = 6 per group). Infected mice (blue) showed significant weight loss compared to PBS-treated controls (red). Data are presented as mean ± SD; **p < 0.01, two-way ANOVA with Bonferroni post-test. **(b)** Representative gross morphology of lungs at 24 h post-infection. Left panel: Lung from a PBS-treated control mouse, showing normal morphology and pink coloration. Right panel: Lung from an S_KLB-infected mouse, exhibiting diffuse edema, increased lobar volume with rounded margins and focal areas of mild consolidation. **(c)** Histopathological analysis of lung tissues by H&E staining. Left panel: Control lung tissue showing normal alveolar architecture with clear alveolar spaces (AS), thin alveolar septa, and intact bronchiolar structures. Right panel: Infected lung tissue demonstrating severe acute pneumonia characterized by alveolar collapse and coalescence, widespread thickening of interalveolar septa due to inflammatory infiltration and edema, dense neutrophilic infiltration in alveolar spaces. **(d)** Detection of cellular apoptosis by TUNEL assay. Upper panel: Control lung tissue showing minimal green fluorescence, indicating negligible apoptotic signal. Lower panel: Infected lung tissue displaying abundant TUNEL-positive apoptotic cells (green fluorescence). Nuclei are counterstained with DAPI (blue). Scale bars: 200 μm. **(e)** Quantitative analysis of apoptotic cells per high-power field (HPF). Data are presented as mean ± SD (*n* = 3); ***p < 0.001, unpaired Student’s *t*-test.

To assess the pathogenicity of strain S_KLB, an acute pulmonary infection model was established in mice via intranasal instillation. At 24 h post-infection, animals in the infected group exhibited pronounced clinical signs, including piloerection, hunched posture, and tachypnea. These clinical manifestations were accompanied by a significant reduction in body weight compared to the control group, as illustrated in Figure 6a. Macroscopic examination following euthanasia revealed normal morphology and pink coloration in control lung tissues. In contrast, lungs harvested from infected mice displayed diffuse edema, increased lobar volume with rounded margins, and focal areas of mild consolidation (Figure 6b).

Histopathological evaluation via hematoxylin and eosin (H&E) staining further delineated the extent of tissue injury (Figure 6c). Control lung tissues maintained normal physiological architecture, characterized by clear alveolar spaces, uniformly thin and well-organized alveolar septa, and intact bronchiolar and vascular structures, with no evidence of inflammatory cell infiltration, exudation, or tissue damage. In stark contrast, infected lung tissues exhibited prominent pathological features consistent with acute pneumonia. Diffuse architectural disruption was evident, manifested by partial alveolar collapse and coalescence, along with widespread thickening of the interalveolar septa attributable to inflammatory infiltration and edema. A dense infiltration of neutrophils was observed diffusely within both alveolar spaces and the pulmonary interstitium. Pulmonary capillaries displayed mild congestion, and proteinaceous eosinophilic exudate was visible within some alveolar lumina. Additionally, partial detachment of bronchiolar epithelial cells with denudation of the basement membrane was noted, alongside marked vascular dilation and congestion.

Apoptosis detection by terminal deoxynucleotidyl transferase dUTP nick end labeling (TUNEL) assay revealed only sporadic apoptotic cells in control lung tissues (Figure 6d). Conversely, infected lung tissues exhibited a substantial increase in the number of TUNEL-positive (green fluorescent) cells. Quantitative analysis confirmed that the apoptotic index was significantly elevated in the infected group compared to controls (Figure 6e).

### 3.4 Impact of S_KLB infection on the expression of inflammatory and oxidative stress-related genes in murine lung tissue

**Figure 7:**
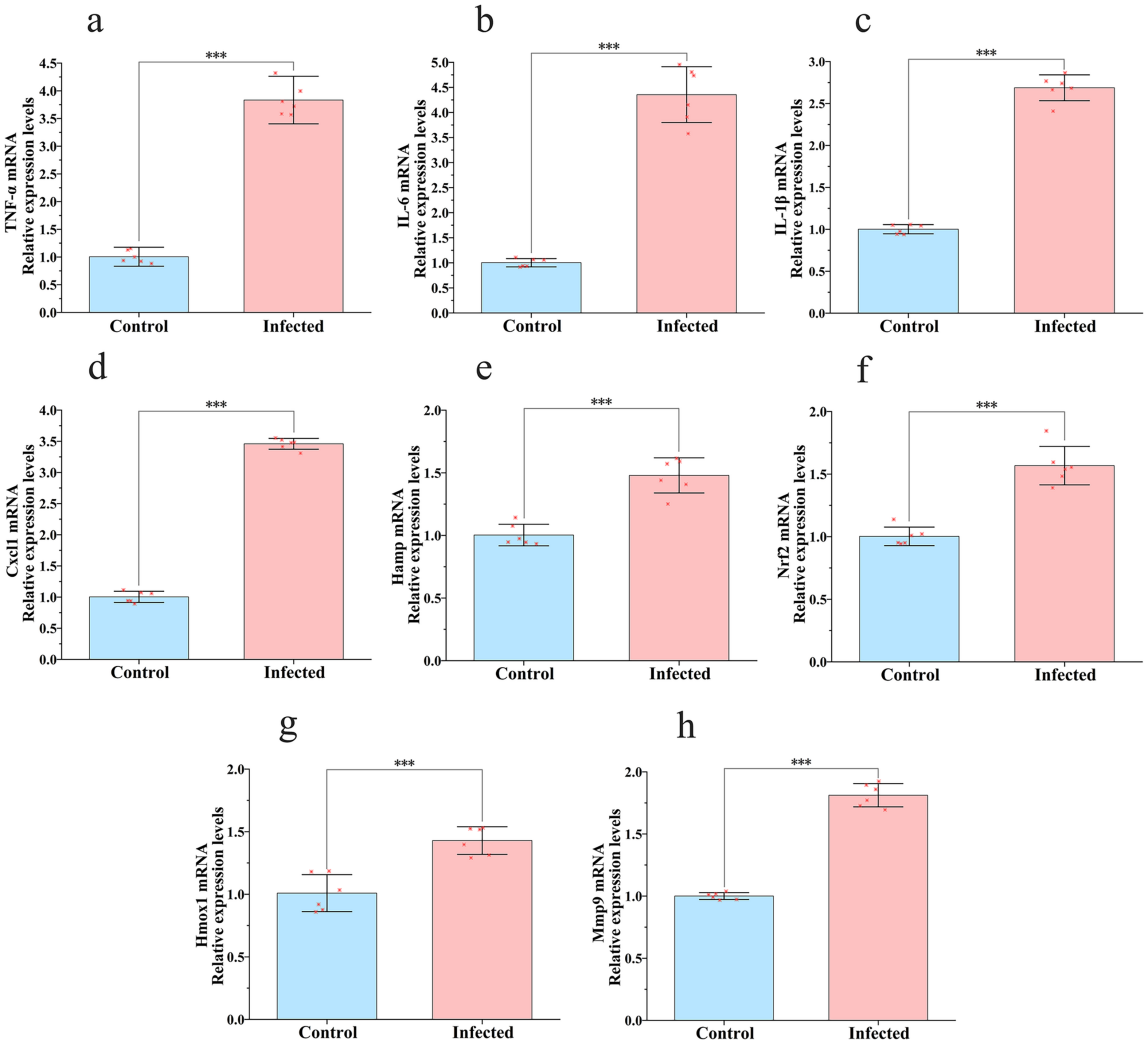
Transcriptional changes of inflammation-, oxidative stress-, and tissue remodeling-associated genes in murine lung tissue following S_KLB_ infection. Gene expression analysis via qRT-PCR showing fold change relative. **(a)** TNFα expression, **(b)** IL6 expression, **(c)** IL1b expression, and **(d)** CXCL1 expression were significantly upregulated, indicating a robust inflammatory response and neutrophil recruitment. **(e)** *Hamp* expression was induced, reflecting host iron-restriction defense. **(f)** Nrf2 and **(g)** *Hmox1* expression were elevated, suggesting activation of the antioxidant defense system. **(h)** *Mmp9* expression was substantially increased, implicating tissue remodeling and injury processes. Data are presented as mean ± SD (*n* = 6 mice per group). Statistical significance was determined by two-way ANOVA with Sidak’s multiple comparisons test: ***p < 0.001.

To elucidate the host molecular response triggered by *K. aerogenes* S_KLB infection, the mRNA expression levels of genes associated with inflammation, oxidative stress, and tissue remodeling were quantified in lung tissues using real-time quantitative PCR, following the approach of Zhang et al. (2025) (Figure 7). The results demonstrated a highly significant upregulation in the mRNA expression of the pro-inflammatory cytokines *Tnfα*, *Il6*, and *Il1b* in infected lungs compared to controls. Concurrently, a marked increase in the expression of the chemokine *Cxcl1* was observed, a finding that aligns with the histopathological observation of extensive neutrophil infiltration.

Regarding iron metabolism regulation, infection significantly induced the expression of *Hamp*, the gene encoding hepcidin, indicating the initiation of a host defense mechanism aimed at restricting pathogen access to essential iron. Within the oxidative stress pathway, the mRNA levels of the core transcription factor *Nrf2* and its downstream target gene *Hmox1* were both significantly elevated in the infected group. This coordinated upregulation suggests activation of the pulmonary antioxidant defense system in response to infection-induced oxidative challenge. Furthermore, the expression of *Mmp9*, a matrix metalloproteinase implicated in tissue remodeling and injury, was also substantially upregulated. This finding may be associated with the alveolar structural destruction observed following infection.

Collectively, these alterations in the transcriptional profile reveal, at the molecular level, a complex host response characterized by robust inflammation, oxidative stress, and concurrent tissue repair and injury processes following S_KLB infection.

### 3.5 Effect of infection on the expression of key signaling pathway proteins in murine lung tissue

**Figure 8:**
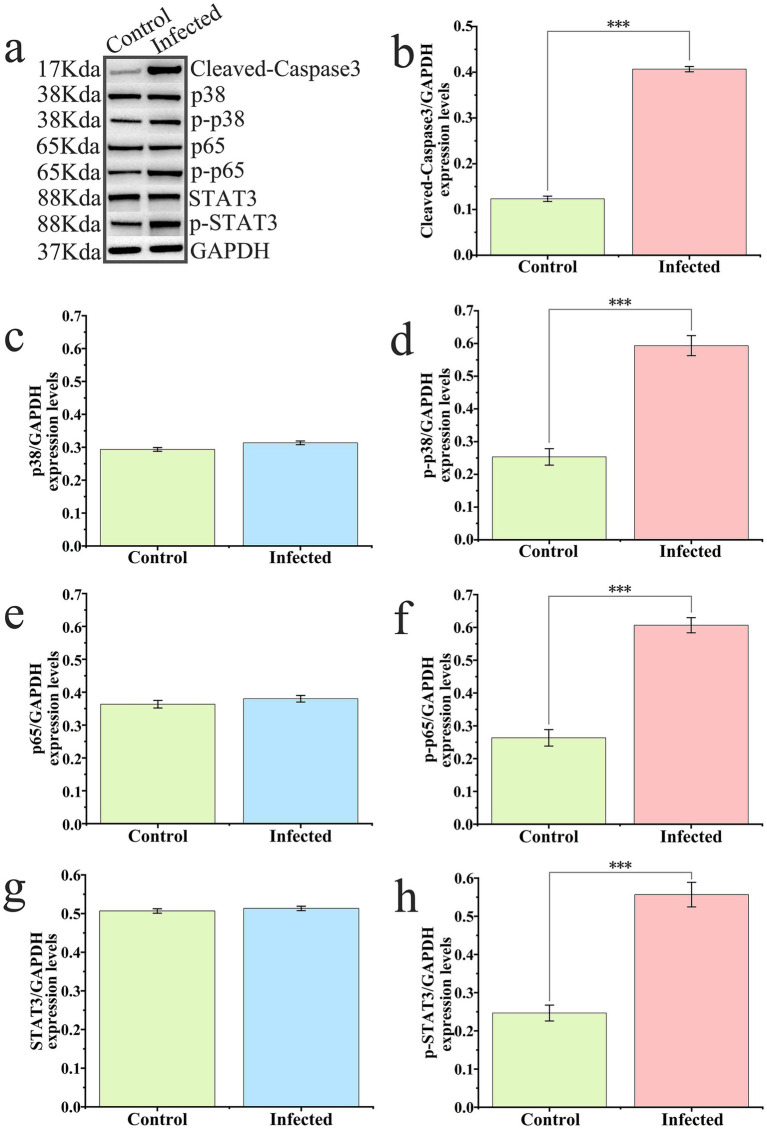
Activation of key signaling pathways and induction of apoptosis in murine lung tissue following S_KLB infection. **(a)** Representative western blots showing protein expression and phosphorylation status in control (Con) and S_KLB-infected (Inf) lung tissue lysates. Proteins were detected in the following order: cleaved Caspase-3, total p38 MAPK, phosphorylated p38 MAPK (p-p38 MAPK), total NF-κB p65, phosphorylated NF-κB p65 (p-NF-κB p65), total STAT3, phosphorylated STAT3 (p-STAT3), and GAPDH (loading control). **(b–h)** Densitometric quantification of protein levels normalized to GAPDH or respective total protein, presented as mean ± SD (*n* = 3 independent experiments). Statistical significance was determined by unpaired Student’s *t*-test: ***p < 0.001. These results demonstrate the concurrent activation of the NF-κB, STAT3, and p38 MAPK signaling pathways, along with enhanced apoptosis execution, in response to S_KLB infection.

To investigate the molecular mechanisms underpinning *K. aerogenes* S_KLB pathogenicity at the protein level, Western blot analysis was performed to assess the phosphorylation status of key proteins within the NF-κB, STAT3, and p38 MAPK signaling pathways, as well as the expression of the apoptosis executioner protein, cleaved Caspase-3 (Figure 8). The results indicated that, compared to the control group, the ratio of phosphorylated NF-κB p65 (p-NF-κB p65) to total NF-κB p65 was significantly increased in the lungs of infected mice, denoting pronounced activation of the NF-κB signaling pathway. Simultaneously, the ratio of phosphorylated STAT3 (p-STAT3) to total STAT3 exhibited a highly significant upregulation, reflecting strong induction of the JAK-STAT3 signaling cascade. Moreover, the ratio of phosphorylated p38 MAPK (p-p38 MAPK) to total p38 MAPK was markedly elevated in the infected group, thereby confirming activation of the p38 MAPK pathway. Regarding apoptosis-associated proteins, the expression level of cleaved Caspase-3 was significantly higher in infected lung tissues compared to controls, a finding that corroborates the increase in cellular apoptosis previously observed via TUNEL staining.

Collectively, these protein-level alterations systematically reveal the molecular pathogenesis of S_KLB infection. The concurrent activation of multiple inflammation- and stress-related signaling pathways—specifically NF-κB, STAT3, and p38 MAPK—appears to drive the abundant production of pro-inflammatory cytokines and ultimately induce pulmonary cellular apoptosis, culminating in severe lung injury.

## 4 Discussion

**Figure 9:**
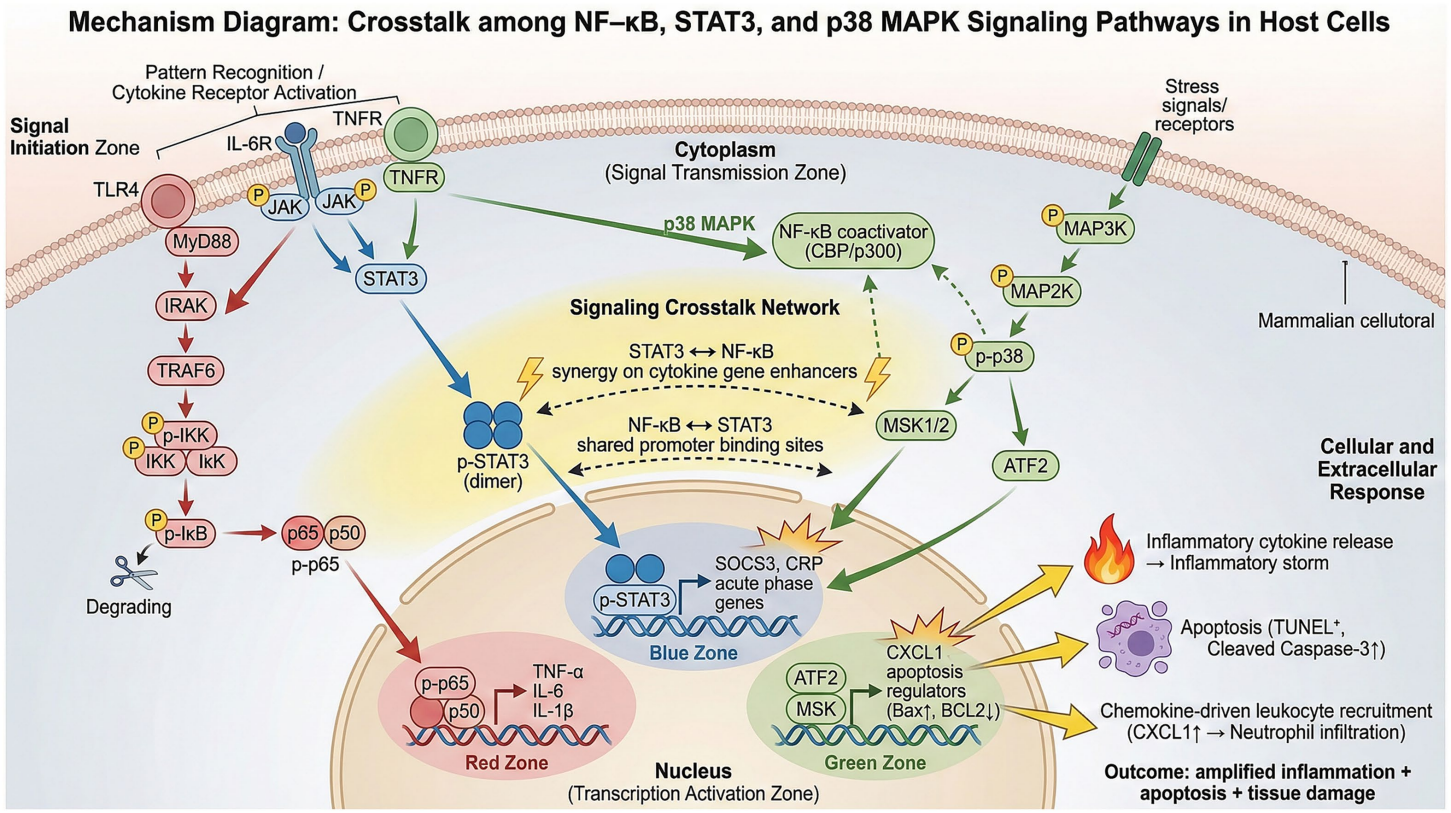
Schematic diagram illustrating the crosstalk among NF-κB, STAT3, and p38 MAPK signaling pathways in host cells.

This study successfully isolated and characterized a *Klebsiella aerogenes* strain, designated S_KLB, originating from the lung of a wild avian host, the ground thrush. Through comprehensive phenotypic and genotypic analysis, combined with a murine infection model and molecular mechanistic investigation, we systematically delineated the multidrug-resistant profile, complete virulence gene repertoire, and the pathogenic mechanism by which this strain induces severe pneumonia through the activation of key signaling pathways (Figure 9).

The growth characteristics of strain S_KLB on various selective media provide initial insights into its ecological adaptability. Its typical lactose-fermenting phenotype on MacConkey agar and robust growth on SS agar not only confirm its taxonomic placement within the *Enterobacteriaceae* family but also suggest a potential tolerance to inhibitory factors such as bile salts, as noted by Kim et al. (2024). This tolerance may be associated with its possession of multidrug efflux pump systems, such as AcrAB-TolC, which are typically involved in extruding antimicrobials, bile salts, and toxic compounds, thereby conferring a significant survival advantage in both host and external environments. Furthermore, growth kinetics analysis revealed that strain S_KLB has a short lag phase (<30 min) and rapid entry into exponential growth in both LB and BHI media, with faster growth in nutrient-rich BHI medium. These characteristics indicate that S_KLB is metabolically adaptable and capable of rapid proliferation under nutrient-rich conditions. During pulmonary infection, inflammation-induced capillary leakage leads to the accumulation of nutrient-rich exudates (including amino acids, carbohydrates, and other metabolites) in the alveolar space. Thus, the nutrient-rich environment of inflamed lung tissue may similarly support rapid proliferation of S_KLB, facilitating the establishment of infection.

Antimicrobial susceptibility testing revealed that S_KLB exhibits resistance to a broad spectrum of agents, including *β*-lactams, macrolides, and select aminoglycosides and fluoroquinolones, presenting a characteristic extensively drug-resistant phenotype. Particularly noteworthy is its resistance to imipenem, a trait often mediated by carbapenemases, which poses a formidable challenge in clinical management, as highlighted by Boattini et al. (2024) and Morgado et al. (2024). This resistance profile aligns with reported trends for multidrug-resistant *K. aerogenes* strains from clinical and environmental sources in recent years. Specifically, Boattini et al. (2024) reported *Klebsiella aerogenes* clinical isolates in Europe show notable carbapenem resistance, while Morgado et al. (2024) identified similar resistance determinants in environmental isolates, suggesting that wildlife-origin strains may harbor resistance elements comparable to those circulating in clinical settings. Notably, the presence of AcrAB efflux pump genes in S_KLB aligns with previous findings linking these systems to both multidrug resistance and enhanced virulence in *K. pneumoniae* (Wan et al., 2022). This congruence suggests that wild birds may serve as reservoirs and potential vectors for antimicrobial resistance genes, and their ecological activities could facilitate the dissemination of resistance, thereby constituting a potential concern for public health surveillance. Future whole-genome sequencing could further elucidate the specific composition of its resistance gene islands and mobile genetic elements to clarify the dissemination potential of its resistance determinants.

Virulence gene profiling revealed that S_KLB possesses a diverse set of virulence associated genes, spanning critical pathogenic stages such as colonization, nutrient acquisition, host defense evasion, and direct tissue damage. The complete presence of siderophore systems (*entB, iroB, iroN*) ensures efficient iron acquisition under the iron-restricted conditions of the host, a pivotal step for successful infection establishment, as discussed by Kumar et al. (2024). Concurrently, the detection of multiple fimbrial adhesin genes (f*imA, mrkA/B*, among others) predicts a strong capacity for adherence and colonization, likely mediating the initial interaction with respiratory mucosal epithelium. The presence of efflux pump genes (*acrA, acrB*), while associated with antimicrobial resistance, may also enhance bacterial survival by extruding host defense molecules such as antimicrobial peptides. This synergistic combination of virulence associated determinants suggests the high pathogenic potential of S_KLB, consistent with the severe infection observed in the murine model. However, it is important to note that PCR detection of these genes indicates presence but does not confirm their expression or functional contribution to pathogenicity; further studies using gene knockout or complementation approaches would be required to establish their specific roles. This comprehensive virulence gene repertoire mirrors that reported for hypervirulent clinical *K. aerogenes* strains; Feng et al. (2024) demonstrated that *K. aerogenes* isolates carrying complete siderophore clusters are associated with increased mortality in murine infection models.

Animal infection experiments directly confirmed the pathogenicity of S_KLB. The acute pneumonic features observed in infected mice—including significant weight loss, pulmonary edema, extensive neutrophilic infiltration, and alveolar structural disruption—closely mirror the pathological alterations commonly associated with Gram-negative bacterial pneumonia. The widespread cellular apoptosis indicated by TUNEL staining further elucidates the cytological mechanism of tissue injury. This study found a dramatic upregulation in the mRNA expression of pro-inflammatory cytokines (*Tnfα, Il6, Il1b*) and the chemokine *Cxcl1* in infected lungs, providing a molecular explanation for neutrophil recruitment and the amplification of the inflammatory cascade. The upregulation of hepcidin (*Hamp*) represents a classic host “nutritional immunity” response aimed at restricting bacterial iron availability, as described by Horváth et al. (2025). The activation of the oxidative stress pathway genes *Nrf2* and *Hmox1* reflects a protective host response against infection-associated oxidative damage, as noted by Seixas et al. (2009) and Sengupta et al. (2022). However, the significant upregulation of *Mmp9* presents a double-edged sword; while it may play roles in tissue repair and inflammation resolution, its overexpression could also exacerbate the degradation of the alveolar basement membrane, thereby promoting the spread of inflammation.

A central mechanistic finding of this study is that S_KLB infection concurrently activated three pivotal intracellular signaling pathways: NF-κB, STAT3, and p38 MAPK (Fann et al., 2018; Fan et al., 2020; Sharata et al., 2025). NF-κB is a canonical pro-inflammatory transcription factor whose activation directly drives the expression of numerous inflammatory cytokines (Fann et al., 2018). STAT3 activation is involved not only in the acute-phase response but also plays complex roles in modulating immune cell function and potentially in suppressing excessive inflammation (Fan et al., 2020). Activation of the p38 MAPK pathway is closely linked to stress responses, cytokine production, and apoptosis (Sharata et al., 2025). The coordinated activation of these three pathways is associated with an intricate signaling network that collectively may contribute to the inflammatory progression and outcome of the infection. Similar concurrent activation of NF-κB, STAT3, and p38 MAPK pathways has been described in response to other Gram-negative pathogens, including *K. pneumoniae* and *E. coli* (Xu et al., 2017; Lin et al., 2024). However, to our knowledge, this is the first report demonstrating simultaneous activation of all three pathways in response to an avian-origin *K. aerogenes* isolate, highlighting the robust inflammatory capacity of this strain. Ultimately, the activation of the downstream apoptosis executioner protein, Caspase-3, correlates with the observed pulmonary cellular apoptosis, suggesting a link between upstream signaling events and tissue injury. While these associations are consistent with a role for these pathways in S_KLB-induced pathology, further functional studies (e.g., using pathway inhibitors or knockout models) would be required to establish causation.

This study provides the first systematic report on the biological characteristics, multidrug resistance profile, comprehensive virulence gene arsenal, and detailed pneumonic pathogenic mechanisms of a wild avian (ground thrush)-derived *K. aerogenes* strain, S_KLB. The findings not only enrich our understanding of the intraspecies genetic and pathogenic diversity of *K. aerogenes* but, more importantly, highlight that wildlife, particularly birds, may represent potential reservoirs for clinically significant drug-resistant and virulent bacterial strains. However, it is important to note that isolation from a deceased bird does not establish causation, and the pathogenic potential demonstrated in the murine model does not directly inform the strain’s role in the original avian host. However, this study has certain limitations. First, 16S rRNA gene identification has limited resolution for closely related Klebsiella species; future confirmation should therefore employ whole-genome sequencing or multilocus sequence typing for more precise speciation. For strain S_KLB, whole-genome sequencing has been completed, and the data are currently being prepared for a separate comparative genomic analysis of avian-origin *Klebsiella* isolates. The 16S rRNA-based identification, supported by phenotypic characterization, is appropriate for the scope of the present study, and the genome data will be made publicly available upon publication of the companion study. Second, the research focused on the acute infection phase (24 h) and did not explore the chronic progression or resolution of the infection. Third, although numerous virulence genes were detected, their specific functional contributions warrant further *in vivo* validation through approaches such as gene knockout experiments. Fourth, antimicrobial susceptibility was assessed using the disk diffusion method, and key findings such as imipenem resistance would benefit from confirmation by MIC-based testing in future studies. Finally, while this study demonstrates the pathogenic potential of an avian-origin *K. aerogenes* isolate in a murine model, it does not provide direct evidence for zoonotic transmission risk. Further studies would be required to assess the strain’s ability to colonize or infect humans or other mammalian hosts under natural conditions.

## 5 Conclusion

This study demonstrates that *Klebsiella aerogenes* strain S_KLB, isolated from the lung tissue of a ground thrush, possesses pathogenic potential in a murine pneumonia model. The strain harbors a diverse set of virulence associated genes, encompassing those involved in siderophore biosynthesis, fimbrial adhesin production, efflux pump systems, and metabolic virulence factors. Furthermore, it is capable of inducing severe pneumonic pathology in mice, which is associated with the activation of multiple key inflammatory signaling pathways, including NF-κB, STAT3, and p38 MAPK. This coordinated activation triggers an excessive host immune-inflammatory response and ultimately culminates in cellular apoptosis. Collectively, these findings elucidate the molecular pathogenesis of this strain in a mammalian model and provide a basis for further investigation into the potential public health implications of wildlife-origin *K. aerogenes* strains.

## Data Availability

The original contributions presented in the study are included in the article/Supplementary material, further inquiries can be directed to the corresponding author.

## References

[ref1] Abbas R. Chakkour M. Zein El Dine H. Obaseki E. F. Obeid S. T. Jezzini A. . (2024). General overview of *Klebsiella pneumonia*: epidemiology and the role of siderophores in its pathogenicity. Biology 13:78. doi: 10.3390/biology13020078, 38392297 PMC10886558

[ref2] Alimu A. Gao Y. Liu J. Lu Y. (2025). Geographic factors influence communities of symbiotic bacterial communities in *Aphis gossypii* across China's major cotton regions. Front. Microbiol. 16:1569543. doi: 10.3389/fmicb.2025.1569543, 40236481 PMC11998284

[ref3] Alonso Villela S. M. Kraïem H. Bouhaouala-Zahar B. Bideaux C. Aceves Lara C. A. Fillaudeau L. (2020). A protocol for recombinant protein quantification by densitometry. Microbiology 9, 1175–1182. doi: 10.1002/mbo3.1027, 32255275 PMC7294310

[ref4] Birgani A. H. Goli H. R. Siadat S. D. Fateh A. Nikbin V. S. Sakhaee F. . (2025). Virulence genes, efflux pumps, and molecular typing of *Klebsiella pneumoniae* isolates from North Iran. AMB Express 15:36. doi: 10.1186/s13568-025-01845-1, 40045145 PMC11883068

[ref5] Boattini M. Bianco G. Llorente L. I. Acero L. A. Nunes D. Seruca M. . (2024). Enterobacterales carrying chromosomal AmpC β-lactamases in Europe (EuESCPM): epidemiology and antimicrobial resistance burden from a cohort of 27 hospitals, 2020-2022. Int. J. Antimicrob. Agents 63:107115. doi: 10.1016/j.ijantimicag.2024.107115, 38367844

[ref6] Chenhaka L. H. Van Wyk D. A. B. Mienie C. Bezuidenhout C. C. Lekota K. E. (2023). The phylogenomic landscape of extended-spectrum β-lactamase producing Citrobacter species isolated from surface water. BMC Genomics 24:755. doi: 10.1186/s12864-023-09867-4, 38062371 PMC10704729

[ref7] Chou T. H. Hu M. H. Hua K. T. Fang C. C. (2025). 2-Chloroethanol induces hepatic toxicity by disrupting endoplasmic reticulum homeostasis ameliorated by dimethyl sulfoxide. Biochim. Biophys. Acta Mol. basis Dis. 1871:168017. doi: 10.1016/j.bbadis.2025.168017, 40818176

[ref8] Duan W. R. Garner D. S. Williams S. D. Funckes-Shippy C. L. Spath I. S. Blomme E. A. (2003). Comparison of immunohistochemistry for activated caspase-3 and cleaved cytokeratin 18 with the TUNEL method for quantification of apoptosis in histological sections of PC-3 subcutaneous xenografts. J. Pathol. 199, 221–228. doi: 10.1002/path.1289, 12533835

[ref9001] Fahim F. J. Prome A. A. Rana S. Uddin M. S. Noor M. Mahfuz S. U. . (2025). Molecular Characterization of β-Lactamase-Resistant Klebsiella aerogenes Isolated from Raw Milk in Bangladesh. Foodborne Pathog Dis. 11, 15353141251372857. doi: 10.1177/15353141251372857, 40892486

[ref9] Fan C. Feng J. Tang C. Zhang Z. Feng Y. Duan W. . (2020). Melatonin suppresses ER stress-dependent proapoptotic effects via AMPK in bone mesenchymal stem cells during mitochondrial oxidative damage. Stem Cell Res. Ther. 11:442. doi: 10.1186/s13287-020-01948-5, 33059742 PMC7560057

[ref10] Fan Y. Mao R. Yang J. (2013). NF-κB and STAT3 signaling pathways collaboratively link inflammation to cancer. Protein Cell 4, 176–185. doi: 10.1007/s13238-013-2084-3, 23483479 PMC4875500

[ref11] Fann D. Y.-W. Lim Y.-A. Cheng Y.-L. Lok K.-Z. Chunduri P. Baik S.-H. . (2018). Evidence that NF-κB and MAPK signaling promotes NLRP inflammasome activation in neurons following ischemic stroke. Mol. Neurobiol. 55, 1082–1096. doi: 10.1007/s12035-017-0394-9, 28092085

[ref12] Feng X. Zhao Y. Yang T. Song M. Wang C. Yao Y. . (2019). Glucocorticoid-driven NLRP3 inflammasome activation in hippocampal microglia mediates chronic stress-induced depressive-like behaviors. Front. Mol. Neurosci. 12:210. doi: 10.3389/fnmol.2019.00210, 31555091 PMC6727781

[ref13] Feng Y. Yang Y. Hu Y. Xiao Y. Xie Y. Wei L. . (2024). Population genomics uncovers global distribution, antimicrobial resistance, and virulence genes of the opportunistic pathogen *Klebsiella aerogenes*. Cell Rep. 43:114602. doi: 10.1016/j.celrep.2024.114602, 39137112 PMC11372444

[ref14] Gibbon M. J. Couto N. Cozens K. Habib S. Cowley L. Aanensen D. M. . (2026). Convergence and global molecular epidemiology of *Klebsiella pneumoniae* plasmids harbouring the IUC3 virulence locus: a population genomic analysis. Lancet Microbe 7:101236. doi: 10.1016/j.lanmic.2025.101236, 41512896

[ref15] Gomez J. C. Dang H. Martin J. R. Doerschuk C. M. (2016). NRF2 modulates host defense during *Streptococcus pneumoniae* pneumonia in mice. J. Immunol. 197, 2864–2879. doi: 10.4049/jimmunol.1600043, 27566827 PMC5026972

[ref16] Hoesel B. Schmid J. A. (2013). The complexity of NF-κB signaling in inflammation and cancer. Mol. Cancer 12:86. doi: 10.1186/1476-4598-12-86, 23915189 PMC3750319

[ref17] Horváth A. Tamási K. Pap R. Jánosa G. Pandur E. (2025). Iron, the essential micronutrient: a comprehensive review of regulatory pathways of iron metabolism. Nutrients 18:109. doi: 10.3390/nu18010109, 41515226 PMC12787992

[ref18] Jones M. R. Quinton L. J. Simms B. T. Lupa M. M. Kogan M. S. Mizgerd J. P. (2006). Roles of interleukin-6 in activation of STAT proteins and recruitment of neutrophils during *Escherichia coli* pneumonia. J. Infect. Dis. 193, 360–369. doi: 10.1086/499312, 16388483 PMC2674298

[ref19] José R. J. Williams A. E. Mercer P. F. Sulikowski M. G. Brown J. S. Chambers R. C. (2015). Regulation of neutrophilic inflammation by proteinase-activated receptor 1 during bacterial pulmonary infection. J. Immunol. 194, 6024–6034. doi: 10.4049/jimmunol.1500124, 25948816 PMC4456635

[ref20] Kim G. E. Ansari S. Andrews G. N. Sasi S. Kolleri J. Abdallah T. A. . (2024). Endogenous purulent pericarditis due to *Klebsiella aerogenes* in a patient with traumatic chest injury: a case report. Cureus 16:e52378. doi: 10.7759/cureus.52378, 38361706 PMC10868625

[ref21] Kumar A. Chakravorty S. Yang T. Russo T. A. Newton S. M. Klebba P. E. (2024). Siderophore-mediated iron acquisition by *Klebsiella pneumoniae*. J. Bacteriol. 206:e0002424. doi: 10.1128/jb.00024-24, 38591913 PMC11112993

[ref22] Kumar V. Chhibber S. (2011). Acute lung inflammation in *Klebsiella pneumoniae* B5055-induced pneumonia and sepsis in BALB/c mice: a comparative study. Inflammation 34, 452–462. doi: 10.1007/s10753-010-9253-9, 20890649

[ref23] Kutilova I. Janecko N. Cejkova D. Literak I. Papagiannitsis C. C. Dolejska M. (2018). Characterization of blaKPC-3-positive plasmids from an *Enterobacter aerogenes* isolated from a corvid in Canada. J. Antimicrob. Chemother. 73, 2573–2575. doi: 10.1093/jac/dky199, 29860384

[ref24] Kong Y. Sun Q. Chen H. Draz M. S. Xie X. Zhang J. . (2021). Transmission dynamics of carbapenem-resistant *Klebsiella pneumoniae* sequence type 11 strains carrying capsular loci KL64 and RMPA/RMPA2 genes. Front. Microbiol. 12:736896. doi: 10.3389/fmicb.2021.736896, 34690977 PMC8529244

[ref25] Laguardia-Nascimento M. de Oliveira A. P. Fernandes F. R. Rivetti A. V. J. Camargos M. F. Fonseca Júnior A. A. (2017). Detection of pseudocowpox virus in water buffalo (*Bubalus bubalis*) with vesicular disease in the state of São Paulo, Brazil, in 2016. Vet. Q. 37, 16–22. doi: 10.1080/01652176.2016.1252479, 27774853

[ref26] Li S. Yu Y. Liu S. Zhang M. Li G. Tong Q. . (2026). Genomic dissection of the clonal background and global dissemination of hypervirulent *Klebsiella pneumoniae* CG23-KL57 lineage. Nat. Commun. 17:1451. doi: 10.1038/s41467-025-68184-4, 41507221 PMC12887077

[ref27] Liu Y. Niu Y. Shan X. Li R. Wang T. Ma X. . (2025). Activating transcription factor 3 alleviates acute liver injury in mice by inhibiting endogenous retroelements. Int. J. Biol. Macromol. 334:149105. doi: 10.1016/j.ijbiomac.2025.149105, 41274477

[ref28] Luo K. Liu M. Peng Z. Zhao H. Li G. Cai Y. . (2025). GNG7 as a tumor-suppressor gene in lung adenocarcinoma: implications for prognosis and immune-based therapies. Front. Oncol. 15:1588646. doi: 10.3389/fonc.2025.1588646, 40496619 PMC12148889

[ref29] Lee D. Oh J. Y. Sum S. Park H. M. (2021). Prevalence and antimicrobial resistance of Klebsiella species isolated from clinically ill companion animals. J. Vet. Sci. 22:e17. doi: 10.4142/jvs.2021.22.e17, 33774933 PMC8007443

[ref30] Lee M. Yi S. Choi J. Park Y. Lim C. Kim Y. (2024). Genomic insights into *Stutzerimonas kunmingensis* TFRC-KFRI-1 isolated from Manila clam (*Ruditapes philippinarum*): functional and phylogenetic analysis. Microorganisms 12:402. doi: 10.3390/microorganisms12122402, 39770605 PMC11677538

[ref31] Li X. Li C. Zhou L. Wang Q. Yao J. Zhang X. . (2024). Global phylogeography and genomic characterization of bla(KPC) and bla(NDM)-positive clinical *Klebsiella aerogenes* isolates from China, 2016-2022. Sci. Total Environ. 923:171560. doi: 10.1016/j.scitotenv.2024.171560, 38458455

[ref32] Li Y. Wang Q. Xiao X. Li R. Wang Z. (2021). Emergence of Bla(NDM-9)-bearing tigecycline-resistant *Klebsiella aerogenes* of chicken origin. J Glob Antimicrob Resist 26, 66–68. doi: 10.1016/j.jgar.2021.04.028, 34051402

[ref33] Lin H. Wieser A. Zhang J. Regel I. Nieß H. Mayerle J. . (2024). Gram-negative bacteria-driven increase of cytosolic phospholipase A2 leads to activation of Kupffer cells. Cell. Mol. Life Sci. 82:22. doi: 10.1007/s00018-024-05451-5, 39725773 PMC11671446

[ref34] Liu W. L. Liu Z. W. Li T. S. Wang C. Zhao B. (2013). Hydrogen sulfide donor regulates alveolar epithelial cell apoptosis in rats with acute lung injury. Chin. Med. J. 126, 494–499. doi: 10.3760/cma.j.issn.0366-6999.20120809, 23422113

[ref35] Matuschek E. Brown D. F. Kahlmeter G. (2014). Development of the EUCAST disk diffusion antimicrobial susceptibility testing method and its implementation in routine microbiology laboratories. Clin. Microbiol. Infect. 20, O255–O266. doi: 10.1111/1469-0691.12373 24131428

[ref36] Mazzon E. Cuzzocrea S. (2007). Role of TNF-alpha in lung tight junction alteration in mouse model of acute lung inflammation. Respir. Res. 8:75. doi: 10.1186/1465-9921-8-75, 17971210 PMC2174464

[ref37] Müller A. Schulze Bernd K. Seinige D. Braun A. S. Kumm F. Kehrenberg C. (2024). Molecular characterization of *Escherichia coli* isolates recovered from broilers with cellulitis. Poult. Sci. 103:103704. doi: 10.1016/j.psj.2024.103704, 38642485 PMC11046064

[ref38] Mani V. Arfeen M. (2024). Betahistine's neuroprotective actions against lipopolysaccharide-induced neurotoxicity: insights from experimental and computational studies. Brain Sci. 14:876. doi: 10.3390/brainsci14090876, 39335372 PMC11430358

[ref39] Mankonkwana B. B. Madoroba E. Magwedere K. Butaye P. (2025). Characterization and antimicrobial resistance of non-Typhoidal Salmonella from poultry carcass Rinsates in selected abattoirs of KwaZulu Natal, South Africa. Microorganisms 13:1786. doi: 10.3390/microorganisms13081786, 40871290 PMC12388589

[ref40] Mark P. J. Wyrwoll C. S. Zulkafli I. S. Mori T. A. Waddell B. J. (2014). Rescue of glucocorticoid-programmed adipocyte inflammation by omega-3 fatty acid supplementation in the rat. Reprod. Biol. Endocrinol. 12:39. doi: 10.1186/1477-7827-12-39, 24886466 PMC4022445

[ref41] Martin G. Tyson G. H. Guag J. Strain E. Ceric O. (2025). Genomic snapshot of Klebsiella spp. isolates from clinically ill animals reveal diverse lineages with limited relatedness to human isolates. BMC Vet. Res. 21:458. doi: 10.1186/s12917-025-04686-z, 40640834 PMC12247286

[ref42] Mei Y. Wang Z. Zhang Y. Wan T. Xue J. He W. . (2019). FA-97, a new synthetic caffeic acid phenethyl ester derivative, ameliorates DSS-induced colitis against oxidative stress by activating Nrf_2_/HO^-1^ pathway. Front. Immunol. 10:2969. doi: 10.3389/fimmu.2019.02969, 31969881 PMC6960141

[ref43] Mo J. Deng Q. Huang Y. Jia X. Xie F. Zhou B. . (2025). Study on the optimization of an extraction process of two triterpenoid saponins in the root of *Rosa laevigata* Michx. and their protective effect on acute lung injury. Pharmaceuticals 18:253. doi: 10.3390/ph18020253 40006066 PMC11859398

[ref44] Morgado S. Fonseca É. Freitas F. Caldart R. Vicente A. C. (2024). In-depth analysis of *Klebsiella aerogenes* resistome, virulome and plasmidome worldwide. Sci. Rep. 14:6538. doi: 10.1038/s41598-024-57245-1, 38503805 PMC10951357

[ref45] Mthembu T. P. Zishiri O. T. El Zowalaty M. E. (2019). Molecular detection of multidrug-resistant salmonella isolated from livestock production systems in South Africa. Infect Drug Resist 12, 3537–3548. doi: 10.2147/idr.S211618, 31814742 PMC6861519

[ref46] Nairz M. Dichtl S. Schroll A. Haschka D. Tymoszuk P. Theurl I. . (2018). Iron and innate antimicrobial immunity-depriving the pathogen, defending the host. J. Trace Elem. Med. Biol. 48, 118–133. doi: 10.1016/j.jtemb.2018.03.007, 29773170

[ref47] Napit R. Gurung A. Poudel A. Chaudhary A. Manandhar P. Sharma A. N. . (2025). Metagenomic analysis of human, animal, and environmental samples identifies potential emerging pathogens, profiles antibiotic resistance genes, and reveals horizontal gene transfer dynamics. Sci. Rep. 15:12156. doi: 10.1038/s41598-025-90777-8, 40204742 PMC11982193

[ref48] Pan F. Xu Q. Zhang H. (2021). Emergence of NDM-5 producing carbapenem-resistant *Klebsiella aerogenes* in a pediatric hospital in Shanghai, China. Front. Public Health 9:621527. doi: 10.3389/fpubh.2021.621527, 33718321 PMC7947282

[ref49] Pollack-Milgate S. Saitia S. Tang J. X. (2024). Rapid growth rate of Enterobacter sp. SM3 determined using several methods. BMC Microbiol. 24:403. doi: 10.1186/s12866-024-03547-3, 39390418 PMC11465882

[ref50] Romero-Durán M. A. Silva-García O. Perez-Aguilar J. M. Baizabal-Aguirre V. M. (2024). Mechanisms of Keap1/Nrf2 modulation in bacterial infections: implications in persistence and clearance. Front. Immunol. 15:1508787. doi: 10.3389/fimmu.2024.1508787, 39763664 PMC11700987

[ref51] Schniering J. Guo L. Brunner M. Schibli R. Ye S. Distler O. . (2018). Evaluation of 99mTc-rhAnnexin V-128 SPECT/CT as a diagnostic tool for early stages of interstitial lung disease associated with systemic sclerosis. Arthritis Res. Ther. 20:183. doi: 10.1186/s13075-018-1681-1, 30115119 PMC6097327

[ref52] Schuetz A. N. Ferrell A. Hindler J. A. Humphries R. Bobenchik A. M. (2025). Overview of changes in the clinical and laboratory standards institute performance standards for antimicrobial susceptibility testing: M100 32nd and 33rd editions. J. Clin. Microbiol. 63:e0162323. doi: 10.1128/jcm.01623-23, 40772786 PMC12421849

[ref53] Seixas E. Gozzelino R. Chora A. Ferreira A. Silva G. Larsen R. . (2009). Heme oxygenase-1 affords protection against noncerebral forms of severe malaria. Proc. Natl. Acad. Sci. USA 106, 15837–15842. doi: 10.1073/pnas.0903419106, 19706490 PMC2728109

[ref54] Sengupta S. Addya S. Biswas D. Banerjee P. Sarma J. D. (2022). Matrix metalloproteinases and tissue inhibitors of metalloproteinases in murine β-coronavirus-induced neuroinflammation. Virology 566, 122–135. doi: 10.1016/j.virol.2021.11.012, 34906793 PMC8648396

[ref55] Seo K. S. Park J. Y. Terman D. S. Bohach G. A. (2010). A quantitative real time PCR method to analyze T cell receptor Vbeta subgroup expansion by staphylococcal superantigens. J. Transl. Med. 8:2. doi: 10.1186/1479-5876-8-2, 20070903 PMC2841588

[ref56] Sharata E. E. Attya M. E. Khalaf M. M. Rofaeil R. R. Abo-Youssef A. M. Hemeida R. A. M. (2025). Levomilnacipran alleviates cyclophosphamide-induced hepatic dysfunction in male Wistar albino rats; emerging role of α-Klotho/TLR4/p38-MAPK/NF-κB p65 and caspase-3-driven apoptosis trajectories. Int. Immunopharmacol. 152:114384. doi: 10.1016/j.intimp.2025.114384, 40056515

[ref57] Song J. Xiang W. Wang Q. Yin J. Tian T. Yang Q. . (2023). Prevalence and risk factors of Klebsiella spp. in milk samples from dairy cows with mastitis-a global systematic review. Front. Vet. Sci. 10:1143257. doi: 10.3389/fvets.2023.1143257, 37035815 PMC10073557

[ref58] Stefanova D. Raychev A. Arezes J. Ruchala P. Gabayan V. Skurnik M. . (2017). Endogenous hepcidin and its agonist mediate resistance to selected infections by clearing non-transferrin-bound iron. Blood 130, 245–257. doi: 10.1182/blood-2017-03-772715, 28465342 PMC5520472

[ref59] Sun D. Haste N. M. Sun J. Serafim M. S. M. Salvioni A. Olson J. . (2025). Repurposing Diflunisal as an Antivirulence agent against *Staphylococcus aureus*. Infect. Microbes. Dis. 7, 43–53. doi: 10.1097/im9.0000000000000174, 40808982 PMC12345599

[ref60] Sun L. Meng N. Wang H. Wang Z. Jiao X. Wang J. (2024). Occurrence and characteristics of bla(OXA-181)-carrying *Klebsiella aerogenes* from swine in China. J. Glob. Antimicrob. Resist. 38, 35–41. doi: 10.1016/j.jgar.2024.04.009 38763331

[ref61] Wan X. Li Q. Olsen R. H. Meng H. Zhang Z. Wang J. . (2022). Engineering a CRISPR interference system targeting AcrAB-TolC efflux pump to prevent multidrug resistance development in *Escherichia coli*. J. Antimicrob. Chemother. 77, 2158–2166. doi: 10.1093/jac/dkac166, 35642356

[ref62] Wang J. Liu Y. Guo Y. Liu C. Yang Y. Fan X. . (2024). Function and inhibition of P38 MAP kinase signaling: targeting multiple inflammation diseases. Biochem. Pharmacol. 220:115973. doi: 10.1016/j.bcp.2023.115973, 38103797

[ref9002] Wang J. Shen Y. C. Chen Z. N. Yuan Z. C. Wang H. Li D. J. . (2019). Microarray profiling of lung long non-coding RNAs and mRNAs in lipopolysaccharide-induced acute lung injury mouse model. Biosci Rep. 39:BSR20181634. doi: 10.1042/bsr20181634, 30979832 PMC6488857

[ref63] Weehuizen T. A. Hommes T. J. Lankelma J. M. de Jong H. K. Roelofs J. J. de Vos A. F. . (2016). Triggering receptor expressed on myeloid cells (TREM)-2 impairs host defense in experimental melioidosis. PLoS Negl. Trop. Dis. 10:e0004747. doi: 10.1371/journal.pntd.0004747, 27253382 PMC4890812

[ref64] Xiao K. Cao Y. Han Z. Zhang Y. Luu L. D. W. Chen L. . (2025). A pan-immune panorama of bacterial pneumonia revealed by a large-scale single-cell transcriptome atlas. Signal Transduct. Target. Ther. 10:5. doi: 10.1038/s41392-024-02093-8, 39757231 PMC11701081

[ref65] Xu G. Wang J. Liu C. H. (2017). *Klebsiella pneumoniae* NdpA suppresses ERK pathway-mediated host early inflammatory responses and is degraded through the ubiquitin-proteasome pathway. Protein Cell 8, 144–148. doi: 10.1007/s13238-016-0341-y, 27844447 PMC5291776

[ref9003] Zhang S. Wen Z. Xu X. Zhou Y. Chen Y. Zhang Y. . (2026). Morus alba L. (Sangzhi) alkaloids alleviate diabetic nephropathy by suppressing endoplasmic reticulum stress and modulating AMPK-associated phosphatidylethanolamine content. Phytomedicine 152:157836. doi: 10.1016/j.phymed.2026.157836 41570782

[ref66] Zhang R. Zheng Y. Ding C. Wu J. Zhu W. Zhu X. . (2025). OxyR contributes to the oxidative stress capacity and virulence of hypervirulent *Klebsiella pneumoniae* ATCC 43816. Front. Cell. Infect. Microbiol. 15:1661384. doi: 10.3389/fcimb.2025.1661384 41574295 PMC12819678

