# Supplementary material for: Characterization and pathogenic mechanisms of a Klebsiella aerogenes strain isolated from a deceased ground thrush
Source: Front Microbiol. 2026 May 12;17:1811142. doi: 10.3389/fmicb.2026.1811142 (PMC13201521; doi:10.3389/fmicb.2026.1811142)
Supplement: Supplementary file 1 [file Data_Sheet_1.PDF]

Supplementary Figures

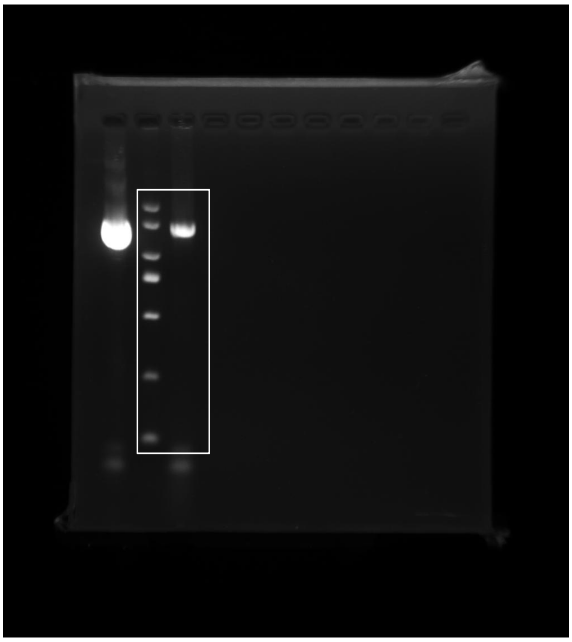

Supplementary Figure 1: Agarose gel electrophoresis of PCR-amplified 16S rRNA gene from strain S_KLB, showing a single specific band of approximately 1500 bp. The framed part in the original image corresponds to the result shown in Figure 3a.

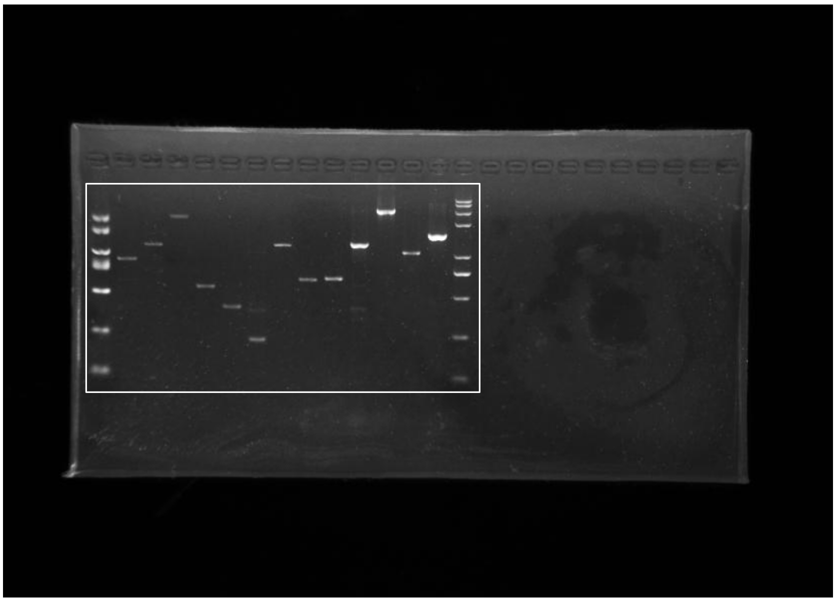

Supplementary Figure 2: Virulence gene profile of *Klebsiella aerogenes* strain S_KLB. Agarose gel electrophoresis showing PCR amplification products of 13 virulence-associated genes from strain S_KLB. The framed part in the original image corresponds to the result shown in Figure 5.

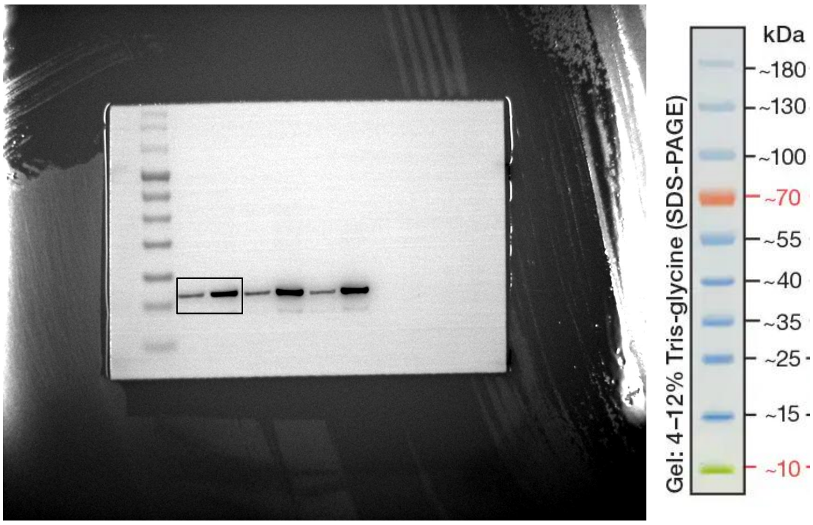

Supplementary Figure 3. Uncropped Western blot image of cleaved Caspase-3 expression in mouse lung tissues. The full, uncropped membrane shows the expression of cleaved Caspase-3 (approximately 17 kDa) in lung tissue lysates from control mice and mice infected with *K. aerogenes* strain S_KLB at 24 hours post-infection. Lanes from left to right contain: pre-stained protein ladder (Marker), followed by three pairs of control (Con) and infected (Inf) samples, representing three biological replicates per group. GAPDH was used as a loading control. The framed part in the original image corresponds to the result shown in Figure 8a.

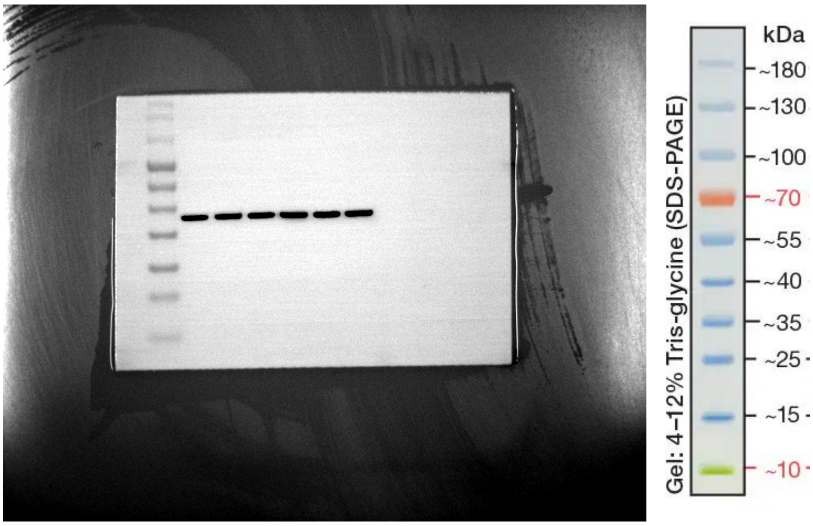

Supplementary Figure 4. Uncropped Western blot image of GAPDH loading control. The full membrane shows GAPDH expression (37 kDa) corresponding to the cleaved Caspase-3 blot in Supplementary Figure 3. Lane order (left to right): Marker, followed by three pairs of control (Con) and infected (Inf) samples. Uniform GAPDH expression confirms equal protein loading.

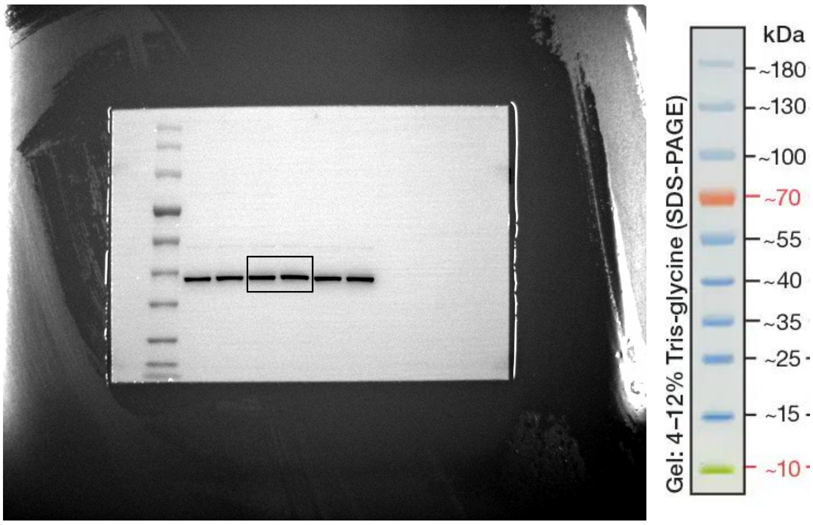

Supplementary Figure 5. Uncropped Western blot image of total p38 MAPK expression in mouse lung tissues. The full, uncropped membrane shows the expression of total p38 MAPK (approximately 38 kDa) in lung tissue lysates from control mice and mice infected with *K. aerogenes* strain S_KLB at 24 hours post-infection. Lanes from left to right contain: pre-stained protein ladder (Marker), followed by three pairs of control (Con) and infected (Inf) samples, representing three biological replicates per group. GAPDH was used as a loading control. The framed part in the original image corresponds to the result shown in Figure 8a.

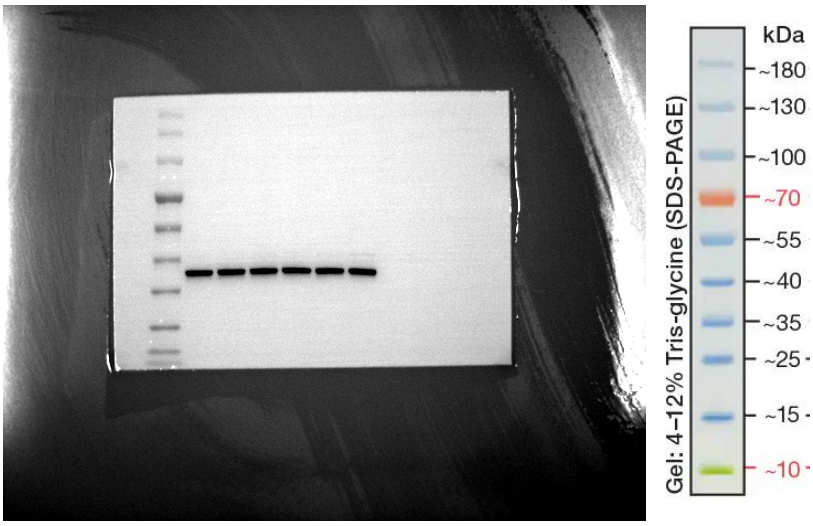

Supplementary Figure 6. Uncropped Western blot image of GAPDH loading control. The full membrane shows GAPDH expression (37 kDa) corresponding to the total p38 MAPK blot in Supplementary Figure 5. Lane order (left to right): Marker, followed by three pairs of control (Con) and infected (Inf) samples. Uniform GAPDH expression confirms equal protein loading.

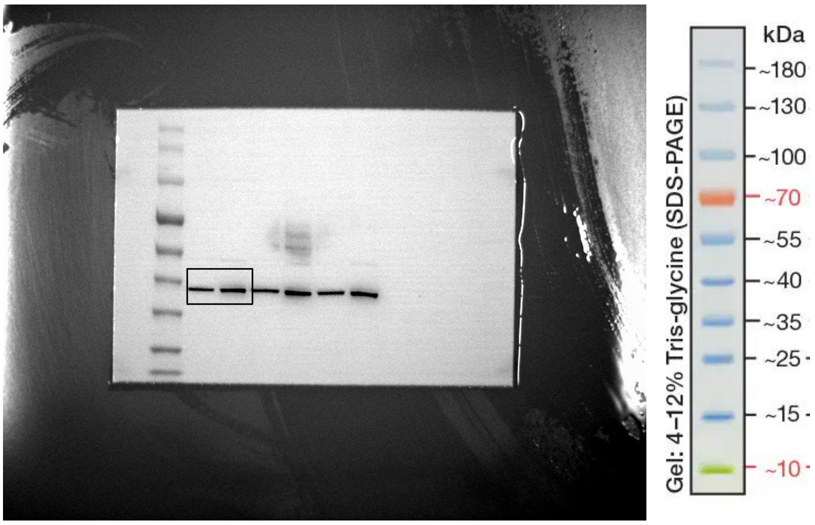

Supplementary Figure 7. Uncropped Western blot image of p-p38 MAPK expression in mouse lung tissues. The full, uncropped membrane shows the expression of p-p38 MAPK (approximately 38 kDa) in lung tissue lysates from control mice and mice infected with *K. aerogenes* strain S_KLB at 24 hours post-infection. Lanes from left to right contain: pre-stained protein ladder (Marker), followed by three pairs of control (Con) and infected (Inf) samples, representing three biological replicates per group. GAPDH was used as a loading control. The framed part in the original image corresponds to the result shown in Figure 8a.

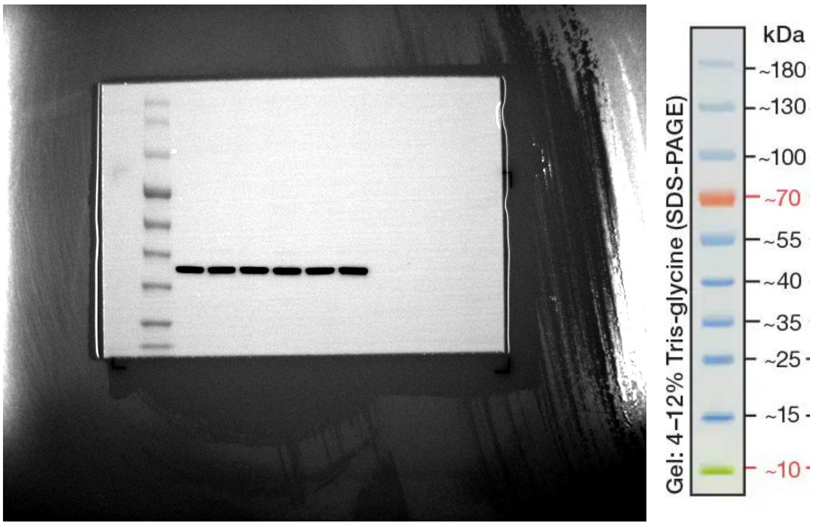

Supplementary Figure 8. Uncropped Western blot image of GAPDH loading control. The full membrane shows GAPDH expression (37 kDa) corresponding to the p-p38 MAPK blot in Supplementary Figure 7. Lane order (left to right): Marker, followed by three pairs of control (Con) and infected (Inf) samples. Uniform GAPDH expression confirms equal protein loading.

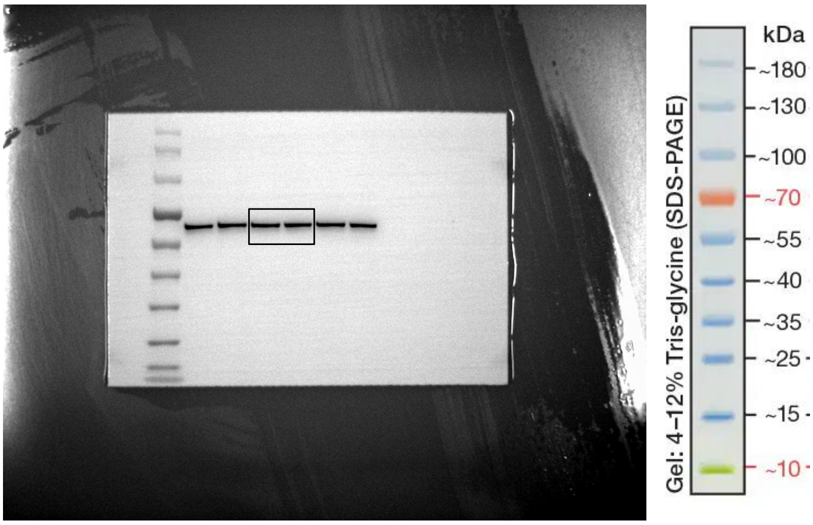

Supplementary Figure 9. Uncropped Western blot image of total p65 expression in mouse lung tissues. The full, uncropped membrane shows the expression of total p65 (approximately 65 kDa) in lung tissue lysates from control mice and mice infected with *K. aerogenes* strain S_KLB at 24 hours post-infection. Lanes from left to right contain: pre-stained protein ladder (Marker), followed by three pairs of control (Con) and infected (Inf) samples, representing three biological replicates per group. GAPDH was used as a loading control. The framed part in the original image corresponds to the result shown in Figure 8a.

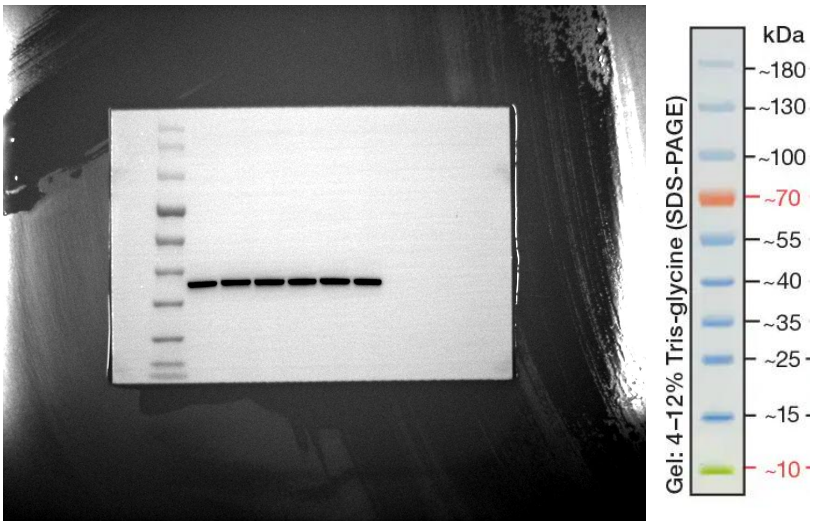

Supplementary Figure 10. Uncropped Western blot image of GAPDH loading control. The full membrane shows GAPDH expression (37 kDa) corresponding to the total p65 blot in Supplementary Figure 9. Lane order (left to right): Marker, followed by three pairs of control (Con) and infected (Inf) samples. Uniform GAPDH expression confirms equal protein loading.

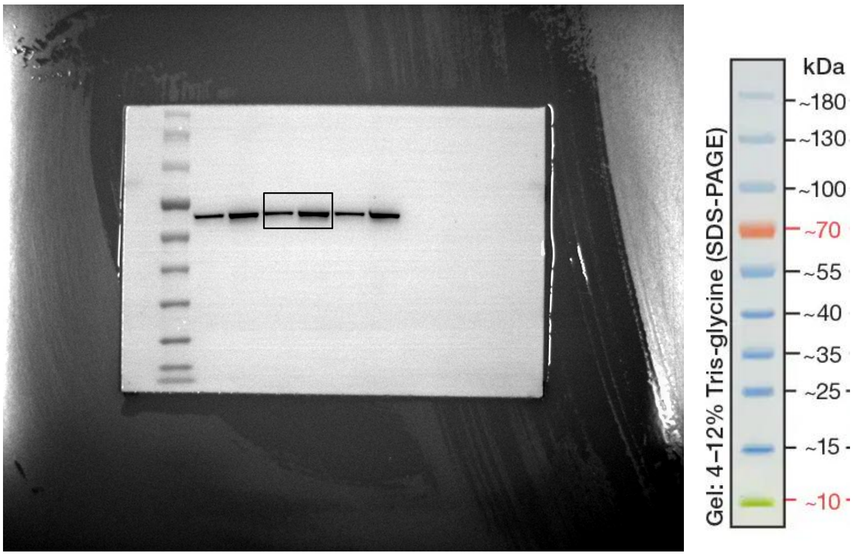

Supplementary Figure 11. Uncropped Western blot image of p-p65 expression in mouse lung tissues. The full, uncropped membrane shows the expression of p-p65 (approximately 65 kDa) in lung tissue lysates from control mice and mice infected with *K. aerogenes* strain S_KLB at 24 hours post-infection. Lanes from left to right contain: pre-stained protein ladder (Marker), followed by three pairs of control (Con) and infected (Inf) samples, representing three biological replicates per group. GAPDH was used as a loading control. The framed part in the original image corresponds to the result shown in Figure 8a.

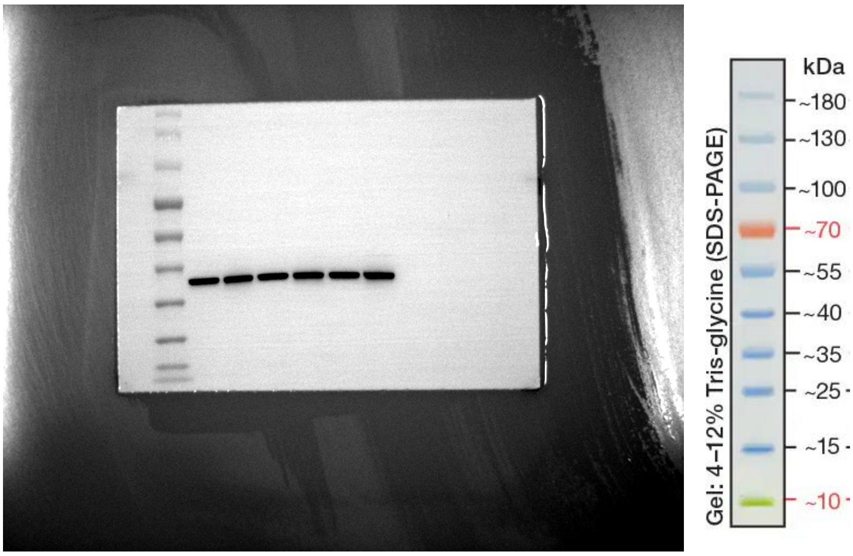

Supplementary Figure 12. Uncropped Western blot image of GAPDH loading control. The full membrane shows GAPDH expression (37 kDa) corresponding to the p-p65 blot in Supplementary Figure 11. Lane order (left to right): Marker, followed by three pairs of control (Con) and infected (Inf) samples. Uniform GAPDH expression confirms equal protein loading.

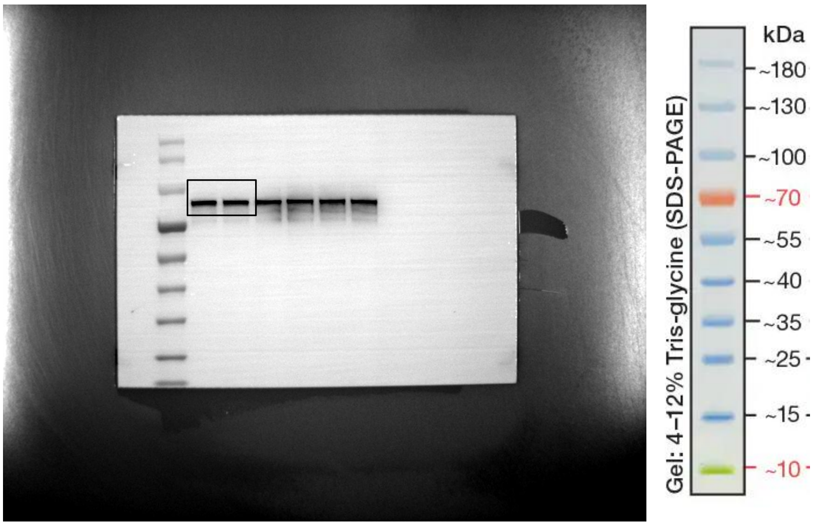

Supplementary Figure 13. Uncropped Western blot image of total STAT3 expression in mouse lung tissues. The full, uncropped membrane shows the expression of total STAT3 (approximately 88 kDa) in lung tissue lysates from control mice and mice infected with *K. aerogenes* strain S_KLB at 24 hours post-infection. Lanes from left to right contain: pre-stained protein ladder (Marker), followed by three pairs of control (Con) and infected (Inf) samples, representing three biological replicates per group. GAPDH was used as a loading control. The framed part in the original image corresponds to the result shown in Figure 8a.

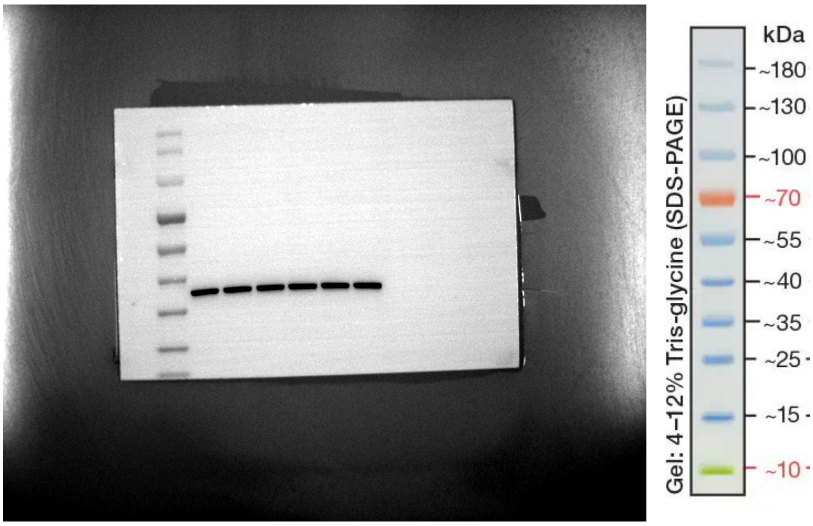

Supplementary Figure 14. Uncropped Western blot image of GAPDH loading control. The full membrane shows GAPDH expression (37 kDa) corresponding to the total STAT3 blot in Supplementary Figure 13. Lane order (left to right): Marker, followed by three pairs of control (Con) and infected (Inf) samples. Uniform GAPDH expression confirms equal protein loading.

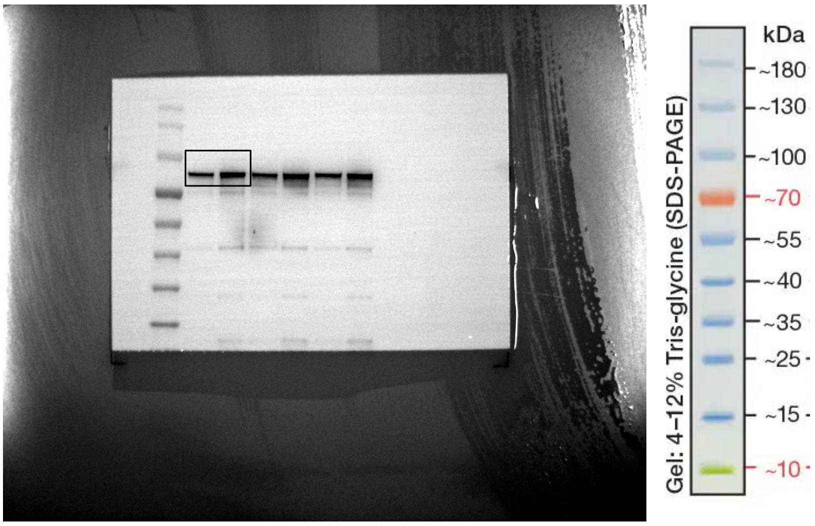

Supplementary Figure 15. Uncropped Western blot image of p-STAT3 expression in mouse lung tissues. The full, uncropped membrane shows the expression of p-STAT3 (approximately 88 kDa) in lung tissue lysates from control mice and mice infected with *K. aerogenes* strain S_KLB at 24 hours post-infection. Lanes from left to right contain: pre-stained protein ladder (Marker), followed by three pairs of control (Con) and infected (Inf) samples, representing three biological replicates per group. GAPDH was used as a loading control. The framed part in the original image corresponds to the result shown in Figure 8a.

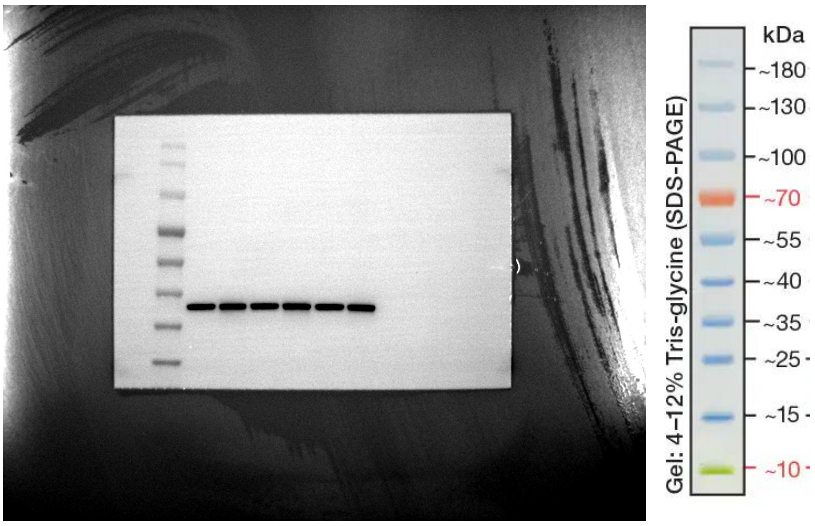

Supplementary Figure 16. Uncropped Western blot image of GAPDH loading control. The full membrane shows GAPDH expression (37 kDa) corresponding to the p-STAT3 blot in Supplementary Figure 15. Lane order (left to right): Marker, followed by three pairs of control (Con) and infected (Inf) samples. Uniform GAPDH expression confirms equal protein loading.
