# Supplementary material for: Characterization and pathogenic mechanisms of a Klebsiella aerogenes strain isolated from a deceased ground thrush
Source: Front Microbiol. 2026 May 12;17:1811142. doi: 10.3389/fmicb.2026.1811142 (PMC13201521; doi:10.3389/fmicb.2026.1811142)
Supplement: Supplementary file 2 [file Table_1.DOCX]

## ****Supplementary Table 1. Primer sequences used for PCR amplification of virulence-associated genes in****Klebsiella aerogenes****strain S_KLB.****

| **Classification** | **virulence genes** | **Pair sequence (5'→3)** |
| --- | --- | --- |
| Iron Acquisition Systems | *entB* | F:TCTAGATTATTTCACCTCGCGGG  R:GAGCTCATGGCAATCCCTAAATTACAGG |
|  | *iroB* | F:TCTAGATTACCCTTTTTGCGCCA  R:GAGCTCGCATTCTGTTTGTTGGCCC |
|  | *iroN* | F:TCTAGAATGAGAATTAACAAGATCCTCTGGCC  R:GAGCTCTCAGAATGTCGCCGTCACTCC |
|  | *fimA* | F:TCTAGATTACTCGTATTGCACTTTGAAGGTGG  R:GAGCTCATGAAAATCAAAACACTGGCAATCG |
|  | *mrkA* | F:TCTAGAATGTCCAGACGCAATAACG  R:GAGCTCGCAGGCGATTAACAACAATAAT |
|  | *mrkB* | F:TCTAGAATGAAATTATTCGCTACCGCCG  R:GAGCTCTTAGTTGTAATACGGTACGCCCAGCTC |
|  | *wecB* | F:TCTAGAGGTAGAAAAACTCATAGCGTTACC  R:GAGCTCGTGAAAGTACTAACTGTATTTGGC |
|  | *rcsA* | F:TCTAGATCAGCGCATATTGACGTAAATGC  R:GAGCTCATGTCAACGATTATCATGGATTTGTGC |
|  | *rcsB* | F:TCTAGAACTCTTTATCCGTTGAGGTAAGAG  R:GAGCTCGAACAATATGAACGTAATTATTGCCG |
| Efflux Pumps & Antimicrobial Resistance | *AcrA* | F:TCTAGAATGAACAAAAACAGAGGGTTAACGCC  R:GAGCTCTTAAGACTTGGTTTGTTCAGACTGAGCG |
|  | *AcrB* | F:GAGCTCGCCTAATTTCTTTATCGATCGCCCCATA  R:GAGCTCTTAATGATGCTCAACCGGATGGTTATGC |
| Toxins | *astA* | F: TCTAGAATGATGGTGATCCGACCG  R: GAGCTCTCATGATGCTTTCTCCTCCGG |
|  | *astD* | F:TCTAGACATGAGTTTATGGATTAACGGCG  R:GAGCTCTCAACTTGCCTGCGATGAAAAATC |

****Supplementary Table 2.** Primers used for qRT-PCR.**

| **Gene** | **Primer sequence (5′→3′)** | **Product size**  **(bp)** |
| --- | --- | --- |
| Tnfa | F: CTTCCAGAACTCCAGGCGGTGC R: TTGGTGGTTTGTGAGTGTGAGGGTC | 184 |
| Il6 | F: GAAATGATGGATGCTACCAAACTG R: ACTCTGGCTTTGTCTTTCTTGTTATC | 139 |
| Il1b | F: TTCAAATCTCGCAGCAGCACATC R: CGTCACACACCAGCAGGTTATCAT | 207 |
| Cxcl1 | F: CCAAACCGAAGTCATAGCCACAC R: TTACTTGGGGACACCTTTTAGCATC | 112 |
| Hamp | F: TGCCTGTCTCCTGCTTCTCCTC R: GCAATGTCTGCCCTGCTTTCTT | 120 |
| Nrf2 | F: TAATACGGAAAACAAGCAGCAGG R: AAGGTGGGATTTGAGTCTAAGGAG | 228 |
| Hmox1 | F: GAAATCATCCCTTGCACGCC R: CCTGAGAGGTCACCCAGGTA | 122 |
| Mmp9 | F: CCCCACTTACTATGGAAACTCAAATG R: CTCAAAGATGAACGGGAACACAC | 232 |
| *GAPDH* | F: CGGTGCTGAGTATGTCGTGGAGTC R: GGCGGAGATGATGACCCTTTTG | 100 |
